# Genome-wide association analyses reveal genomics basis for frizzled feather and candidate genes associated with growth traits in Yuexi frizzled feather chicken

**DOI:** 10.1186/s12711-026-01107-z

**Published:** 2026-11-12

**Authors:** Sucheng Peng, Zihao Zhang, Zengguang Liao, Sadaqat Ali, Jin Guo, Yubo Zhou, Shudai Lin, Li Zhang

**Affiliations:** 1 College of Coastal Agricultural Sciences, Guangdong Ocean University, Zhanjiang, 524088 China; 2 College of Food Science and Engineering, Guangdong Ocean University, Zhanjiang, 524088 China; 3 Guangdong Engineering Research Center of Livestock and Poultry Bio-breeding, Zhanjiang, China

## Abstract

**Background:**

Yuexi Frizzled Feather Chicken (YFC), an indigenous chicken breed in Western Guangdong of China (Yuexi region), is well known for its great adaptability and excellent tolerance to the high temperature and high humidity climates due to its frizzled feather characteristics. In order to investigate the genomic structure and genetic markers of frizzled feather in YFC, we conducted a genome wide analysis study (GWAS) for feather types using the whole genome resequencing data from 356 YFC and 85 non-frizzled feather chickens.

**Results:**

Selective sweep analyses using Fst, ROD, and XPCLR were performed and a total of 267 overlapping genes were identified. Overlapping genes were subjected to GO and KEGG enrichment analysis, indicating that keratin filament, positive regulation of collateral sprouting, and positive regulation of protein kinase B signaling might be related to the frizzled feather formation. Further investigation by GWAS identified *KRT5 *and *KRT3 *as important linked genes, with sanger sequencing revealing one SNP and one InDel in*KRT5*, as well as one SNP in *KRT3 *could be used as markers for frizzled feather characteristics. Although supplementary experiments targeting the 15‑bp deletion in *KRT75L4 *revealed that *KRT5 *and *KRT3 *are not the true causal genes but are merely in linkage disequilibrium with the causal variant, these association signals still provide valuable clues for the genetic dissection of the frizzle feather phenotype in this breed. Given genome-wide studies on body weight and body size traits of the YFC breed are limited, GWAS was also conducted on YFC for growth traits. The results demonstrated several genes associated with growth traits of YFC, including body weight (*ERC1*,*RAD52*,*ATP8A2*,*PPARGC1A*), keel length (*RFX4*,*TMPRSS15*,*INPP4B*,*LUZP2*,*CCNJL*,*IGF1*), body slope length (*RBFOX1*), tibia length (*IGF2BP1*), tibia circumference (*SCRN1*,*ERO1B*,*RAB4A*,*MAPK10*), chest width (*MID1*,*GRID1*,*ARHGAP6*,*FRMPD4*), and chest depth (*LOC121109148*).

**Conclusion:**

Our studies not only provide new insights into the haplotype background and genetic variation landscape of the frizzled feather trait, but also identify several genes associated with growth traits that might contribute to genetic improvement of chickens.

**Supplementary Information:**

The online version contains supplementary material available at https://doi.org/10.1186/s12711-026-01107-z.

## Background

Through history, hundreds of chicken breeds have evolved through the combined effects of natural and artificial selection [1], leading to significant phenotypic variations in plumage type, body size, egg color, and flying ability [2]. As one of the most sophisticated integumentary systems in birds, feathers are characterized by their intricate hierarchical branching patterns and outstanding biomechanical properties [3]. Feather morphogenesis is controlled through the coordinated interaction of temporal and spatial mechanisms [4]. Distinct from other branching structures in nature, the formation of a feather initiates from the distal end, progresses proximally to form a series of hierarchical appendages that anchor into the skin [5]. The domestic chicken exhibits an extraordinary degree of phenotypic variation, representing the most phenotypically diverse avian species, with particularly diversity in feather type [4]. Elucidating the genetics basis of feather variability in chicken could provide insights into the evolutionary diversification in morphological traits during adaptation or selection. Feathers, as the most notable appearance features of poultry, exhibit a high degree of diversity, such as smooth feathers [6], silky feathers [7], and frizzled feathers [8]. Therefore, the formation of feather phenotypes reflects the complex genetic variations and underlying regulatory mechanisms that have evolved over time to adapt to local environmental conditions.

Western Guangdong of China (Yuexi region) features a prolonged hot and humid climate, which brought a huge challenge to the heat dissipation in poultry. Under heat stress conditions, poultry typically experience significant mortality rates and severe declines in production performance. As birds lack sweat glands, their ability to adapt to high temperatures is severely compromised, particularly with high humidity [9]. Therefore, the negative impacts of high ambient temperatures and the hazards of heat stress pose numerous challenges to the growth and development of chickens [10, 11]. Yuexi Frizzled Feather Chicken (YFC), an indigenous Chinese breed characterized by the outward curling of feather rachises, barbs, and partially exposure of skin, is a high-quality germplasm resource with advantages of high heat resistance, strong adaptability, and tolerance to roughage. With its excellent heat dissipation, robust disease resistance, and good meat quality, YFC is well-adapted to the hot and humid climate in Western Guangdong of China and favored by the local consumers and the poultry industry. YFC presents a valuable model for investigating the genetic basis of evolutionary adaptations, heat dissipation, and stress resistance, given its unique feather morphology and robust environmental adaptability.

Frizzled feather is a highly heritable trait that follows Mendelian inheritance [8]. Previous molecular studies have explored the signals and progresses in development, regeneration, and evolution of feathers through localized expression patterns [12]. Early studies indicated that *KRT6A* (designated as *KRT75* in the original publication) is likely associated with the frizzled feather phenotype [4], while the *KRT75L4* was also found to be correlated with the frizzled feather trait [8, 13]. Advances in sequencing technologies have enabled highly efficient and precise genome-wide detection of genetic variants in livestock and poultry. In particular, the advancement of high-throughput sequencing has been of great benefit to the analysis of genomics structure and genetic diversity, leading to the promotion of molecular breeding in livestock and poultry [14]. Whole genome resequencing, a genome-wide detection technology for high-density single nucleotide polymorphisms (SNPs) and insertion-deletion variants (InDels), has facilitated the comprehensive investigation of population genetic architecture, environmental adaptation mechanisms, and evolutionary dynamics underlying unique indigenous breeds of chickens [15, 16]. The diverse and complex forms of feathers offer an ideal model for investigating the development and evolution of morphology. Existing chicken feather mutants are particularly valuable for pinpointing the genetic factors that control feather formation. Thus, with the great potential of whole genome resequencing, more distinctive genetic characteristics and evolutionary mechanisms underlying the YFC can be discovered.

Genome-wide studies on body weight and body size traits of the YFC breed are limited, which prompted us to investigate such morphological traits. Growth traits represent one of the key target traits in molecular breeding programs for poultry, as they directly impact production efficiency and economic value. Growth traits, as complex quantitative traits governed by polygenic inheritance, present significant challenges for conventional breeding approaches to achieve substantial improvements [17, 18]. Genome-wide association studies (GWAS) have successfully identified multiple candidate genes related to body weight and size traits in poultry. For example, *RELT* and *FAM168A* in Chinese yellow-feathered chickens [19], and *FOXO1*, *KPNA3*, *DGKH*, *DLEU7*, *CAB39L*, *KCNIP4*, and *SLIT2* in a F2 resource population derived from a cross between the broiler and Baier yellow chicken have all been linked to body weight (BW) traits [20]. Additionally, numbers of studies had revealed candidate genes for body size traits, including for body slope length (BSL), keel length (KL), tibial length (TL), tibial circumference (TC), chest width (CW), and chest depth (CD) [17, 21–24]. Therefore, this study employed GWAS to identify genomic loci associated with growth traits in poultry, thereby revealing the genetic mechanisms regulating chicken growth and carcass traits and providing potential targets for molecular breeding strategies.

In this study, YFC chickens were subjected to whole-genome resequencing and genetic analyses to elucidate the genetic basis underlying frizzled feather were performed, along with a GWAS for body weight and size traits in this breed. Our findings can provide a reference for future breeding efforts in this local population.

## Materials and methods

### Sample collection and DNA extraction

In this study, a total of 356 YFCs were collected from the Gaozhou Wannong Yuexi Frizzled Feather Chickens breeder farm (Additional file 1: Figure S1) [25]. All YFC individuals were from the same batch and raised under standardized management conditions in the same poultry house, with ad libitum access to feed and water, and under identical light programs. A 1 mL whole blood sample was collected from each chicken in 0.5 M EDTA and stored at −20 °C. High-quality genomic DNA was extracted from blood samples using the FastPure Blood/Cell/Tissue/Bacteria DNA Isolation Mini Kit (DC112-01, Vazyme Biotech Co., Ltd, Nanjing, China). Quality and concentration of DNA were quantified through the 1% gel electrophoresis and the Ultra-micro spectrophotometer (Nanodrop Lite, Thermo scientific).

For Sanger sequencing and genome wide analysis of feather types, the genomic DNA of 25 Qiandongnan Xiaoxiang chickens (QXC) and 60 Qibainong chickens (QC) were collected (Additional file 1: Figure S1). For Sanger sequencing to validate the polymorphic loci, the genomic DNA of non-frizzled chickens were collected, including 30 Bantam chickens (BC), 30 Wenchang chickens (WC), 30 Huaixiang chickens (HC), 30 Qingyuan Ma chickens (QMC), and 30 Huxu chickens (HXC) (Additional file 1: Figure S1).

### Whole genome sequencing and annotation

Whole genome resequencing was conducted on YFC and QC, with the reference genome of GRCg7b version (https://ftp.ensembl.org/pub/release-112/fasta/gallus_gallus/dna/). Calling of SNPs and InDels for YFC and QC were performed on the DNBSEQ platform (BGI, Shenzhen, China) and the Illumina platform (Majorbio, Shanghai, China), respectively. Detailed information for sequencing data is provided in the supplementary materials (Additional file 2: Table S1 and Additional file 3: Table S2).

Whole genome resequencing data of QXC was publicly available and downloaded for subsequent analysis [15] (Additional file 4: Table S3), and SNP and InDel calling were performed through the following steps. Raw reads were subjected to filtration using the SOAPnuke v1.5.6 [26] software to eliminate adapter sequences and reads of poor quality, which included reads with a quality score below 20, an N ratio exceeding 10%, and low-quality surpassing 50%. The software BWA v0.7.17 [27] was applied to map the cleaned data onto the reference genome GRCg7b. Subsequently, Samtools v1.2 [28] was employed to organize the mapped reads based on their genomic coordinates, with the final results stored in BAM format files. The GATK 3.3 [29] variant caller software was employed to call SNPs and InDels from the BAM files, adhering to best-practice recommendations. To keep exclusively high-quality SNPs and InDels, the following filtering criteria were applied: for SNPs, “Quality by Depth (QD) <2.0 || Fisher Strand (FS) >60.0 || root mean square of Mapping Quality < 20.0 || MQRankSum < −12.5 || Strand Odds Ratio (SOR) >3.0 || ReadPosRankSum < −8.0”; and for InDels, “QD < 2.0 || FS > 200.0 || SOR > 10.0 || MQRankSum < −12.5 || ReadPosRankSum < −8.0”.

To gain a more comprehensive understanding and analysis of the genomic characteristics for YFC, the VCFtools [30] software was used to integrate the resequencing data of YFC with that of QC and QXC.

### Measurement of growth traits

Measurements of growth traits were conducted on 356 YFCs (313 females, 43 males) at the age of 140 days in accordance with the PRC’s Poultry Production Performance Terminology and Measurement Statistical Methods “NY/T823-2004”, including keel length (KL), body slope length (BSL), tibial length (TL), tibial circumference (TC), chest width (CW), and chest depth (CD). The measurement methods were as follows: BSL was quantified as the distance between shoulder joint and ischial tuberosity, while KL was quantified as the distance from front-to-end of the keel process. TL was determined as the straight distance between the superior tibial joint and the third/fourth toes. CD was measured from the first thoracic vertebra to the anterior edge of the keel. CW was measured as the distance between the two shoulder joints. In addition, the body weight of YFCs was measured using a weighing scale.

In this study, GWAS was performed to screen for candidate genes associated with growth traits using 313 female individuals from YFC. Descriptive statistics for the measured growth traits were calculated for the 313 female chickens.

### Genetic relationship analysis

The decay of linkage disequilibrium (LD) was evaluated by calculating the squared correlation (*r*^2^) between any pair of loci using PopLDdecay v3.41 [31]. To prepare for principal component analysis (PCA) of SNP genotypes, we applied LD-based SNP pruning using PLINK v1.9 [32], with parameters (–indep-pairwise 50 10 0.2) to remove redundant loci. For PCA, the first two principal components were visualized using the ggplot2 package in R. Phylogenetic relationships were inferred by constructing a neighbor-joining tree (NJ tree) by using VCF2Dis [33], and the NJ tree was visualized using the iTOL (version 4) [34] online tool.

### Genome-wide selective sweep analysis

Whole genome resequencing data of YFC, QC, and QXC were classified into 2 distinct groups according to the feather type, frizzled chicken (YFC) and non-frizzled chicken (QC, QXC), to explore selective sweep regions associated with the development of curly feather in YFC. Selective sweeps were detected using the fixation index (Fst) [35], reduction of diversity (ROD) [36], and cross-population composite likelihood ratio (XPCLR) methods [37]. Fst was estimated using VCFtools, ROD was derived from π ratio reduction, and XPCLR analyses were conducted with XPCLR v1.0 [37]. The Fst, ROD, and XP-CLR were calculated with 100-kb windows and slides with 10-kb steps.

Windows that coincided and ranked in the top 5% for Fst, ROD, and XPCLR were collectively considered as candidate regions that have been under selection, in order to mitigate the arbitrariness of a single threshold and control false positives effectively. It should be noted that the top 5% cutoff was employed as an initial screening strategy to prioritize regions for further investigation, rather than as an indicator of statistically significant selective sweeps.

### Genome-wide association studies

Genome-wide association study (GWAS) was performed in YFC to further support results from the selection sweep analysis. A univariate mixed linear model (MLM) in EMMAX [38] was applied to identify SNPs and InDels significantly associated with growth and feather shape traits. The models are as follows:

1 $${\bf{y}} = {\bf{X\alpha }} + {\bf{Z\beta }} + {\bf{W\mu }} + {\bf{e}},$$

where **y** represents a vector comprising observed phenotype trait values; **Xα** represents the fixed effect vector, which includes corrections for population stratification and fixed effects such as candidate SNPs or InDels as covariates; **Zβ** denotes the SNP or InDel effect vector, encompassing allelic substitution effects **β**; **Wμ** represents random animal effects, with the variance-covariance structure based on the kinship correlation matrix estimated from whole genome SNP or InDel genotypes; and **e** is a vector of random residuals. Manhattan and Quantile-Quantile plots were generated utilizing the R package.

All chromosomes were included in the analysis, but this study focused primarily on autosomal loci. Accordingly, no sex-chromosome-specific adjustments were applied, and the Z chromosome was analyzed using the same model as the autosomes. Associations on the Z chromosome should therefore be interpreted with caution and are not included among the main conclusions.

For growth traits, we constructed a genome-wide kinship matrix using the EMMAX software [38], which quantified the genetic relatedness among all 313 female YFC individuals. This matrix was incorporated into the random effect (**Wμ**) of the MLM model, which effectively corrects for the false positive signals caused by familial genetic relatedness (a core component of population structure) in the population.

Significance thresholds were adjusted by means of the Bonferroni test and quality control (–maf 0.05 –geno 0.1) was adjusted via PLINK v1.9 [32] software. For SNPs, the suggestive significance threshold was set at -log10(*P*)=7.10, and the genome-wide significance threshold at -log10(*P*)=8.10. For InDels, the suggestive significance threshold was set at -log10(*P*)=6.14, and the genome-wide significance threshold at -log10(*P*)=7.44. Due to the limitations of the current sample size, we additionally adopted an exploratory threshold (-log10(*P*) = 5) that has been used in other similar studies within the field [39]. This approach was intended to make full use of the available data and provide valuable clues for future research.

Heritability estimates for growth traits were computed as the ratio of the genetic variance component to the total phenotypic variance, expressed as:

2 $${{\bf{\it{h}}}^2} = {{{{\bf{\it{\sigma }}}^2}_{\bf{\it{g}}}} \over {{{\bf{\it{\sigma }}}^2}_{\bf{\it{g}}} + {{\bf{\it{\sigma }}}^2}_{\bf{\it{e}}}}},$$

where σ^2^_g_ represents the genetic variance estimated from the kinship matrix and σ^2^_e_ denotes the residual variance, as derived from the mixed linear model implemented in EMMAX.

### GO and KEGG analysis

The KOBAS 3.0 online (http://bioinfo.org/kobas/genelist/) platform was utilized for Kyoto Encyclopedia of Genes and Genomes (KEGG) pathway and gene ontology (GO) enrichment analyses. Using a significance threshold of *p* < 0.05, we identified significantly enriched GO terms and KEGG pathways and visualized the results with R scripts.

### SNP and InDel genotyping by Sanger sequencing

Polymerase Chain Reaction (PCR) and Sanger sequencing were conducted to confirm the SNP or InDel found to be associated with frizzled feather trait. To validate the candidate loci for frizzled feather trait, chickens five non-frizzled chicken breeds, were used(Additional file 1: Figure S1). PCR primers were designed by Oligo7 software (version 7.56, Molecular Biology Insights, US) and synthesized by Sangon Biotech (Guangzhou, China) (Additional file 5: Table S4). Genotyping of the samples was performed by direct sequencing (Sangon Biotech, Guangzhou, China) using Applied Biosystems ABI 3730 L Genetic Analyzer (Applied Biosystems group, US), and the results were analyzed by SnapGene software (GSL Biotech LLC, Chicago, IL).

To verify the presence of the previously reported 15 bp deletion in *KRT75L4*, specific primers flanking the deletion site were designed (Additional file 5: Table S4), and PCR amplification was performed on five samples from our study group (one YFC and four HC individuals).

## Results

### Analysis of genetic relationships among different feather types

**Fig. 1:**
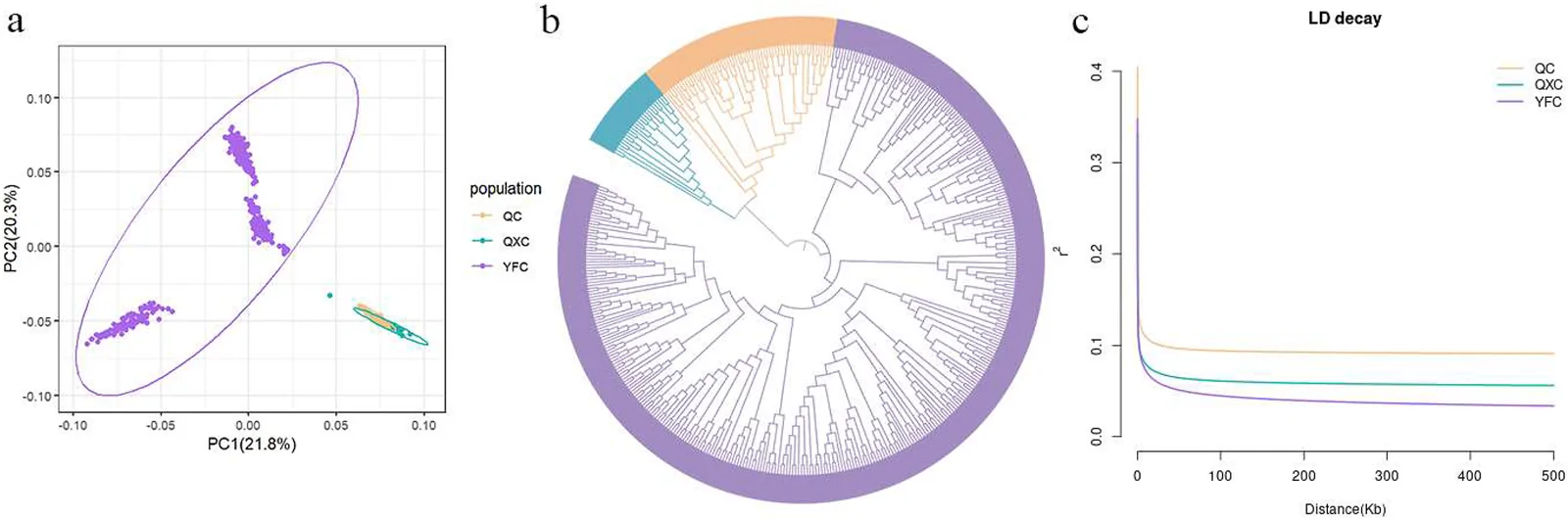
Population genetic analysis between YFC and non-frizzled chicken breeds. (**a**) Principal component analysis plot with three chicken breeds. (**b**) Neighbor-joining tree constructed by genetic distance among three chicken breeds. (**c**) Plots of squared allele frequency correlations (*r*^*2*^) by physical distance between sites in three chicken breeds. YFC: yuexi frizzled feather Chicken; QC: Qibainong Chicken; QXC: Qiandongnan Xiaoxiang chicken

In total, 21,925,850 high-quality SNPs and 4,590,765 InDels were obtained from the resequencing data of the 356 YFC individuals [25], and 11,531,107 SNPs and 1,444,166 InDels were obtained from the resequencing data of the 60 QC individuals, with whole genome resequencing data of 25 QXCs downloaded from the public database [15]. In order to study the genetic relationships between frizzled chicken and non-frizzled chickens, we investigated the population structure of YFC, QC, and QXC using PCA and Maximum Likelihood methods. The PCA results showed the first 2 principal components accounted for 21.8 and 20.3% of the total variation (Fig. 1a) and revealed distinct clustering between YFC and non-frizzled chicken (QC and QXC), with QC and QXC showing partial overlap while maintaining separable clusters (Fig. 1a), highlighting the specific differences between YFC and non-frizzled chickens. Furthermore, NJ trees constructed from genome-wide SNPs showed that individuals from the same chicken breed constituted similar clusters, indicating a high level of genetic similarity within each breed (Fig. 1b). Notably, substantial genetic differences were observed between frizzled feather YFC chicken and non-frizzled chickens, with YFC forming a monophyletic clade, while QC and QXC comprising a shared branch with internal subclustering (Fig. 1b). The result of NJ tree analysis was fully consistent with the PCA patterns, possibly due to distinctive genetic variation, suggesting divergence between frizzled feather chickens and non-frizzled chickens.

Linkage disequilibrium (LD) decay analysis provided further insights into the genetic diversity of each chicken breed. Compared to the non-frizzled chickens, the YFC exhibited lower *π* values and faster LD decay rates, indicating reduced genetic diversity and higher reorganization rate (Fig. 1c).

### Genome-wide scan for selective sweeps associated with frizzled feather phenotype

**Fig. 2:**
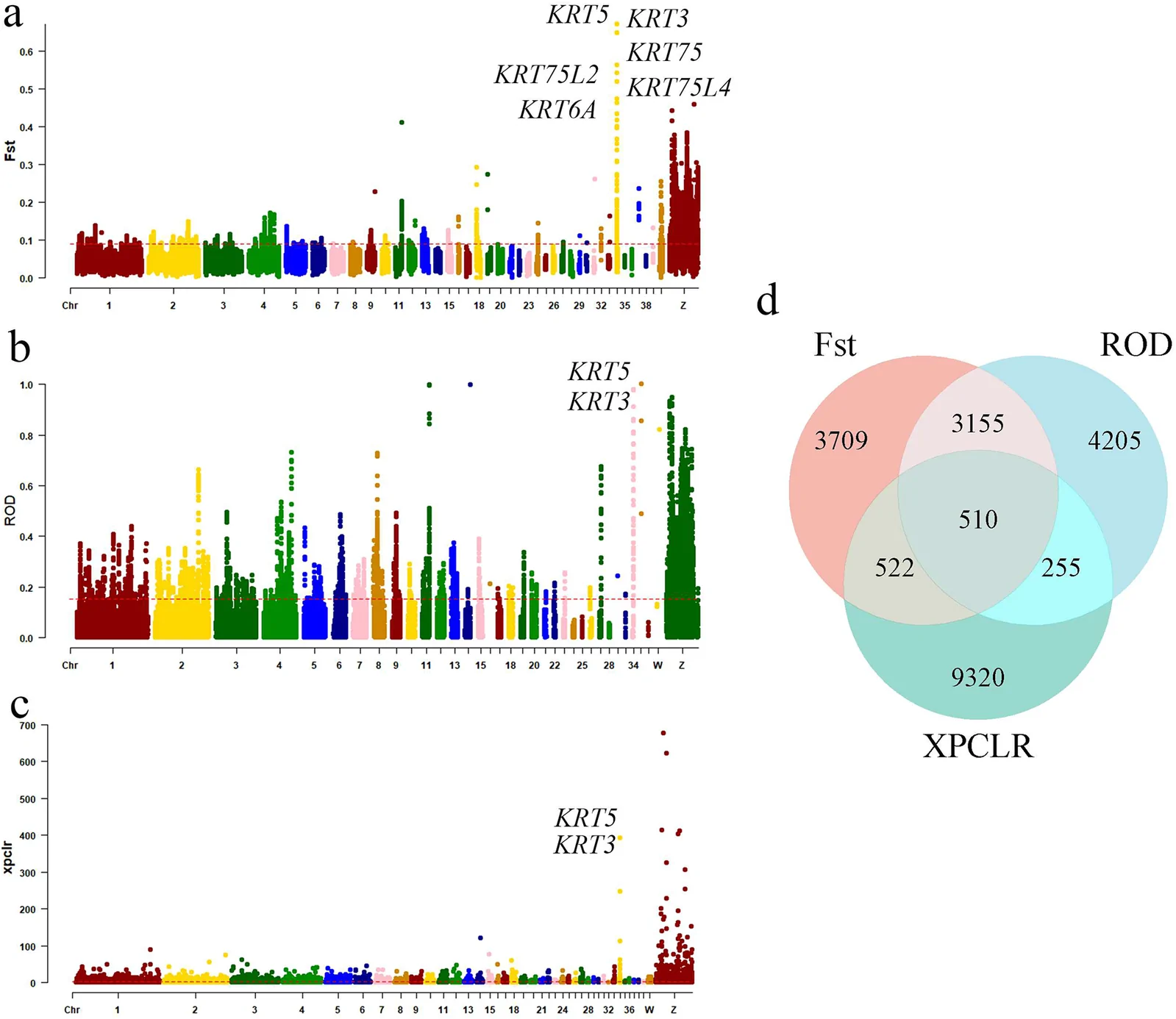
Detection of selective sweep windows in YFC. (**a**) Genome wide distribution of Fst. (**b**) Genome wide distribution of ROD. (**c**) Genome wide distribution of XPCLR. The red dash line indicates the top 5% threshold. (**d**) The overlapping area of the three selection scanning methods

Using YFC as the test group and QC and QXC as the control groups, we identified unique genomic signatures specific to YFC. Genomic regions with values ranking in the top 5% were classified as potential regions of interest. A total of 7896, 8127, and 20,249 candidate regions were identified based on Fst, ROD, and XP-CLR, respectively (Fig. 2 a-c). A Venn diagram identified 510 overlapping selected regions among those identified using Fst, ROD, and XP-CLR, with 267 genes identified and subsequently annotated (Fig. 2dand Additional file 6: Table S5). Notably, *KRT5* (keratin 5) was identified as one of the most strongly selected genes and received particular attention, as its mutation in humans was associated with structural hair abnormalities [40].

### Enrichment analysis of candidate genes in selective sweep regions

**Fig. 3:**
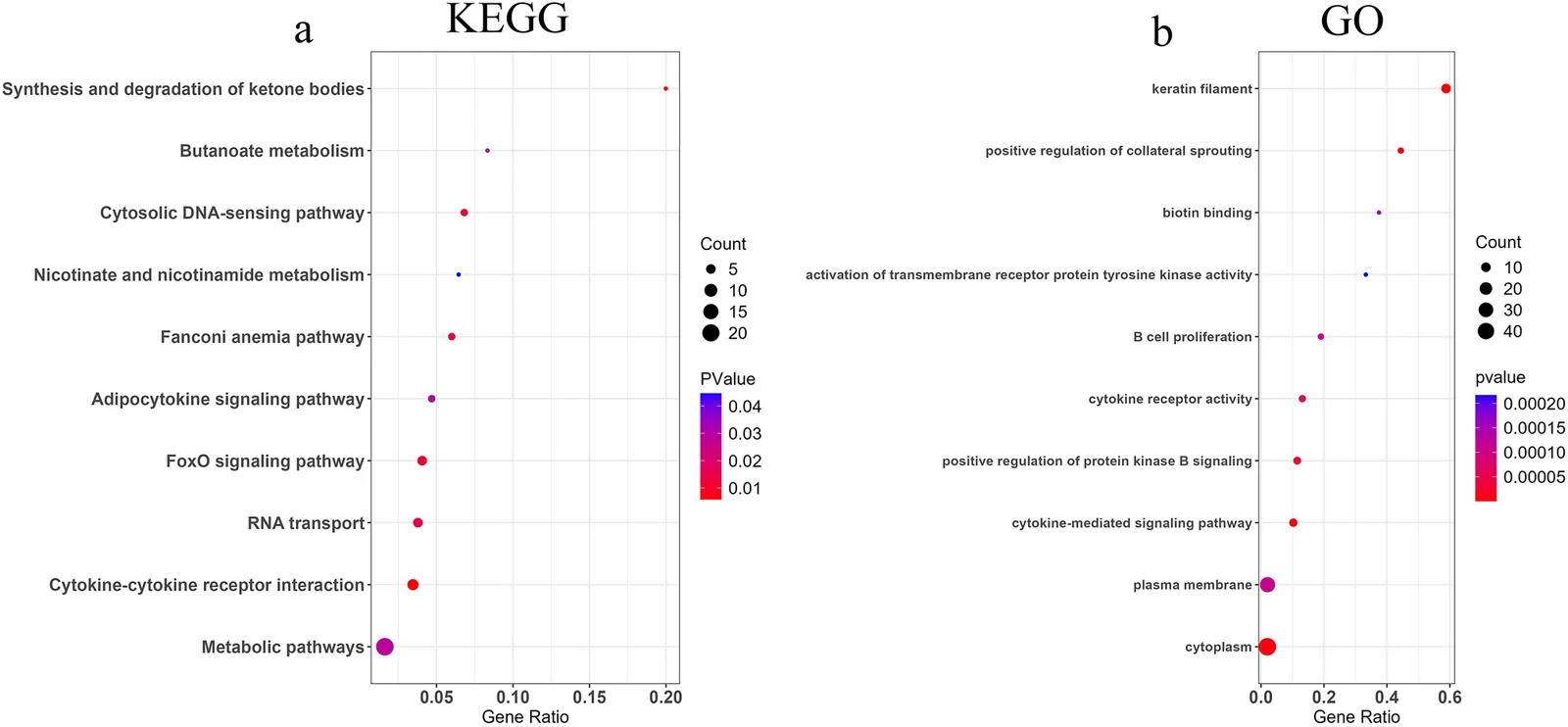
Functional enrichment analysis of candidate genes associated with the frizzle feather trait. (**a**) Bubble chart illustrating the Kyoto encyclopedia of genes and genomes (KEGG) pathway analysis results. (**b**) Bubble chart illustrating the gene ontology (GO) enrichment analysis results

The 267 genes identified in the regions through the overlapping Venn diagram among the three methods were subjected to GO and KEGG enrichment analyses, revealing 10 significantly enriched pathways and 150 GO terms (Additional file 7: Table S6). Several pathways associated with important economic traits were observed through KEGG, including Cytokine-cytokine receptor interaction (gga04060), Cytosolic DNA-sensing pathway (gga04623), and Fanconi anemia pathway (gga03460), which were involved in Disease resistance breeding-related pathways (Fig. 3a), as well as Synthesis and degradation of ketone bodies (gga00072), FoxO signaling pathway (gga04068), and Adipocytokine signaling pathway (gga04920), which were associated with growth performance-related pathways (Fig. 3a). Moreover, Metabolic pathways (gga01100) and Butanoate metabolism (gga00650) linked to meat quality improvement and feed efficiency-related pathways were also enriched (Fig. 3a). Several terms related to feather-associated functions were identified in the GO enrichment (Fig. 3b). Positive regulation of collateral sprouting (GO:0048672) and positive regulation of protein kinase B signaling (GO:0051897) were potentially involved in feather regeneration and growth efficiency (Fig. 3b). Specifically, keratin filament (GO:0045095), enriched with genes such as *KRT5*, *KRT6A*, *KRT75*, and *KRT75L4*, may be associated with feather curling (Fig. 3b) [5], indicating the roles in these genes to functionally regulate plumage structure development.

### Genome wide association study of frizzled feather traits

**Fig. 4:**
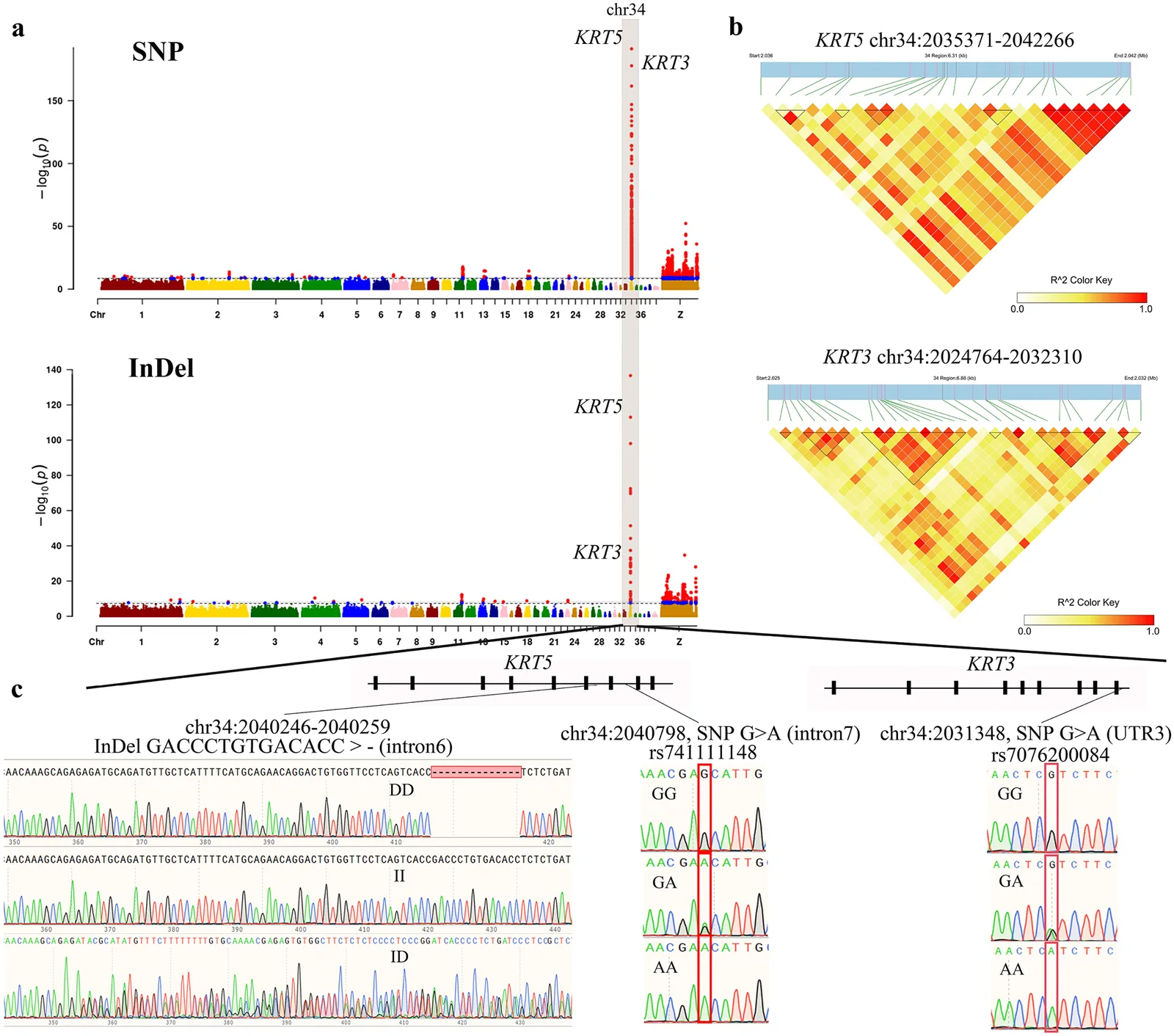
Genome wide association study (GWAS) for frizzled feather trait. (**a**) GWAS for frizzled feather trait in YFC based on SNP and InDel data. (**b**) Linkage disequilibrium plot of variant loci in the *KRT5* and *KRT3* genes on chromosome 34. (**c**) Structure diagram of *KRT5* and *KRT3* gene with black bars representing exons as well as Sanger sequence traces of one InDel and two SNP loci

We then performed GWAS using the frizzled feather group as the test group and the non-frizzled feather group as the control to identify candidate genes associated with frizzled feather formation in YFC. Gene annotation of the loci was performed through the NCBI database. GWAS results revealed two suggestive significant peaks on chromosomes 34 and Z, containing 2859 suggestive significant SNPs and 351 suggestive significant InDels, highlighting the potential candidate genes involved in the frizzled feather trait (Fig. 4a). The associated Q-Q plot authenticated the accuracy of the GWAS findings (Additional file 8: Figure S2). The most suggestive significant loci were on the genes *KRT5* and *KRT3*, which overlapped the top 5% windows detected in the selection signal scan of YFC against other non-frizzled chickens. Additionally, the LD block diagram of the *KRT5* and *KRT3* genes showed strong SNP linkage, indicating a potential high correlation between these genes and the frizzled feather phenotype (Fig. 4b). Therefore, *KRT5* and *KRT3* were prioritized as key candidate genes due to their significant correlation with the frizzled feather trait.

### Sanger sequencing of the variant loci located on the *KRT5, KRT3* and *KRT75L4* genes

To further verify the polymorphism of *KRT5* and *KRT3* and their association with the frizzled feather trait, PCR and Sanger sequencing were conducted on frizzled feather chickens (YFC) and non-frizzled feather chickens (QC, BC, WC, HC, QMC, HXC). One SNP (g.2040798 G > A, rs741111148) and InDel (g.2040242_2040255delCACCGACCCTGTGA) with significantly low *p*-values in the *KRT5* gene were selected for further validation. Sanger sequencing confirmed the presence of the SNP (g.2040798 G > A, rs741111148) and InDel (g.2040242_2040255delCACCGACCCTGTGA) in the *KRT5* (Fig. 4c), but the actual locus of the Indel in YFC was confirmed to be as follows: g.2040246_2040259delGACCCTGTGACACC.

The genotyping result showed that YFC are all homozygous for deletion at Indel (g.2040246_2040259delGACCCTGTGACACC), while other normal feathered chicken breeds are almost exclusively homozygous wild type or heterozygous for the deletion, except for the HC breed, in which two individuals were found to be homozygous for the deletion (Table 1). As for SNP (rs741111148), only YFC have the AA genotype, while other non-frizzled feather breeds have either GG or GA genotypes. Therefore, by using these two loci in combination, we could identify the frizzled feather chicken breed.

**Table 1:** Sanger sequencing statistics for the three loci located in the *KRT5* and *KRT3* genes

Breeds	Phenotype	*KRT5*-InDel			*KRT5*-SNP (rs741111148)			*KRT3*-SNP (rs7076200084)		
		II	ID	DD	GG	GA	AA	GG	GA	AA
YFC(*n* = 90)	frizzle	0	0	90	0	0	90	0	0	90
QC(*n* = 30)	normal	18	12	0	28	2	0	29	1	0
BC(*n* = 30)	normal	26	4	0	30	0	0	30	0	0
WC(*n* = 30)	normal	15	15	0	28	2	0	30	0	0
HC(*n* = 30)	normal	19	9	2	28	2	0	30	0	0
QMC(*n* = 30)	normal	14	16	0	30	0	0	30	0	0
HXC(*n* = 30)	normal	21	9	0	30	0	0	30	0	0

II: homozygous wild; ID: heterozygous deletion; DD: homozygous deletion. YFC: Yuexi frizzled feather chicken; QC: Qibainong chicken; BC: Bantam chicken; WC: Wenchang chicken; HC: Huaixiang chicken; QMC: Qingyuan Ma chicken; HXC: Huxu chicken

The frizzled feather trait is a complex phenotype that is likely influenced by multiple genes. Hence, we also performed Sanger sequencing validation on the candidate SNP located in the *KRT3* gene (Fig. 4c). At rs7076200084, all YFC individuals exhibited the mutant genotype, whereas chickens from the normal-feathered breeds were either homozygous or heterozygous for the reference allele (Table 1). These results further confirmed that *KRT5* and *KRT3* were crucial for the formation of the frizzled feather trait.

*KRT5* and *KRT3* are actually very close to the causal variant in *KRT75L4* (15 bp deletion in exon 3) reported in an earlier study [13]. To investigate the presence of the previously reported 15 bp deletion in *KRT75L4*, we designed specific primers flanking the deletion site and performed PCR amplification using five samples from our study group, comprising one YFC chicken and four HC chickens. Sanger sequencing revealed that the 15 bp deletion was indeed present in the YFC individual, whereas it was absent in all HC samples (Additional file 9: Figure S3). This indicates that the 15 bp deletion in *KRT75L4* was indeed present in the YFC breed, and the variants at *KRT3* and *KRT5* were in strong linkage disequilibrium with this deletion.

### Description of growth traits in female YFCs

**Fig. 5:**
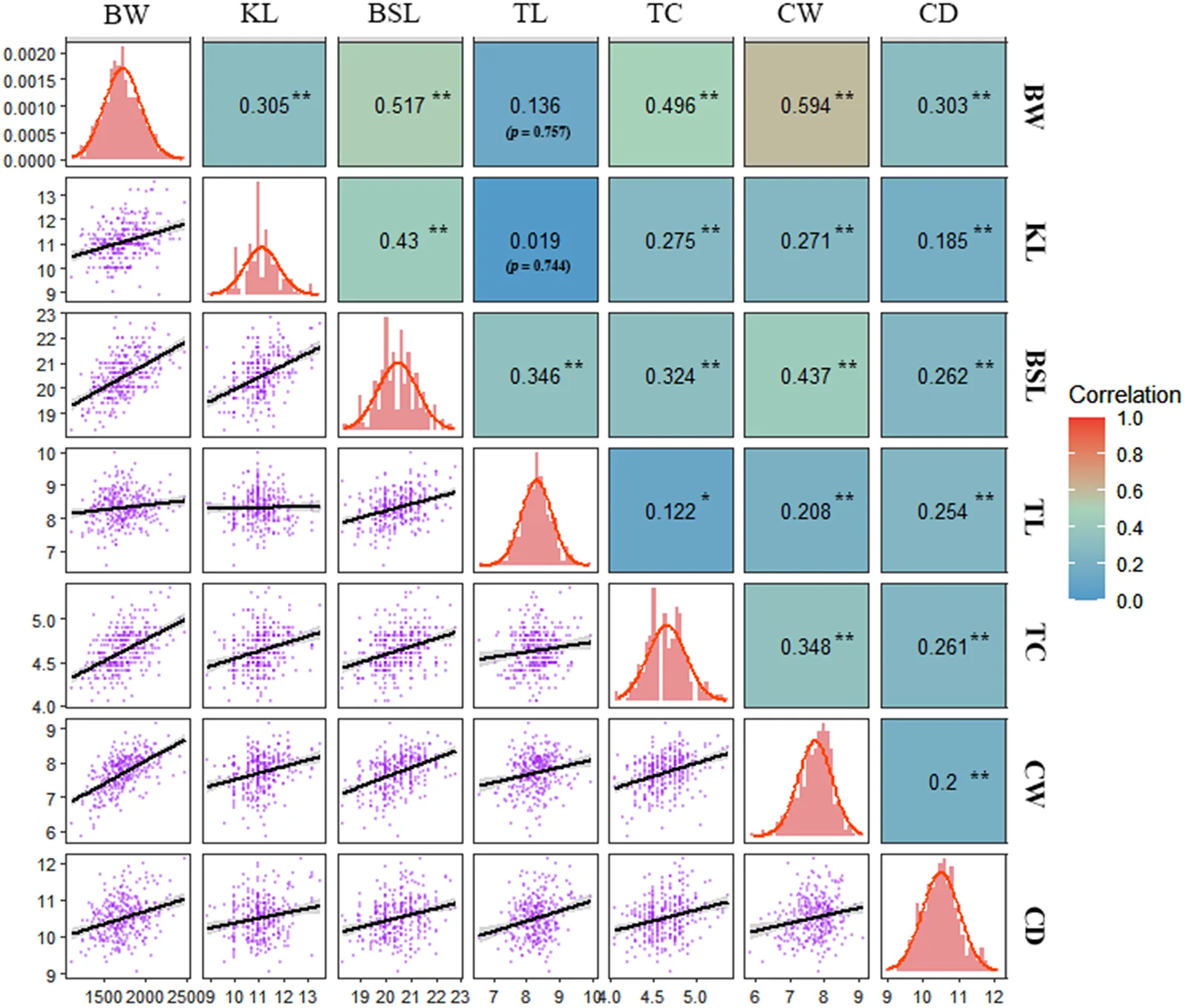
Correlation analysis of body weight and size traits in YFC. The upper right side shows correlations between different traits, the diagonal side shows the distribution of each trait, and the lower left side shows the dispersion. BW: body weight, KL: keel length, BSL: body slope length, TL: tibial length, TC: tibial circumference, CW: chest width, CD: chest depth. *p < 0.05. **p < 0.01

Given the scarcity of genome-wide research on YFC body weight and size traits, we conducted a further analysis of its morphological traits through GWAS. The description of the seven growth traits, including BW (body weight), keel length (KL), body slope length (BSL), tibial length (TL), tibial circumference (TC), chest width (CW), and chest depth (CD), is shown in Table 2. The phenotypic values of all traits exhibited approximately normal distributions (diagonal side of Fig. 5), with skewness and kurtosis values close to zero (absolute values < 1), indicating that the phenotypic values are suitable for GWAS analysis (Table 2).

**Table 2:** Descriptive information of body weight and size traits in 313 female individuals from YFC breed

Trait	Mean	Sd^1^	Median	Min	Max	Skew	Kurtosis	Se ^2^	h2
Body Weight (g)	1745.38	234.38	1730.90	1112.50	2482.30	0.244	0.004	13.25	0.469
Keel Length (cm)	11.08	0.74	11.00	8.90	13.50	0.259	0.871	0.04	0.635
Body Slope Length (cm)	20.47	0.82	20.40	18.30	22.80	0.089	−0.029	0.05	0.535
Tibial Length (cm)	8.32	0.48	8.32	6.56	9.97	0.050	0.624	0.03	0.750
Tibial Circumference (cm)	4.63	0.23	4.60	4.05	5.35	0.237	0.203	0.01	0.366
Chest Width (cm)	7.71	0.52	7.78	5.84	9.14	−0.525	0.477	0.03	0.556
Chest Depth (cm)	10.51	0.53	10.51	9.05	12.09	0.239	0.046	0.03	0.590

^1^Statistical standard deviation. ^2^Standard error of statistics

Using the delta values derived from EMMAX, the heritability estimates for the seven growth traits (BW, KL, BSL, TL, TC, CW, CD) were 0.469 (0.020), 0.635 (0.024), 0.535 (0.022), 0.750 (0.020), 0.366 (0.017), 0.556 (0.022), and 0.590 (0.023), respectively. These moderate-to-high heritability estimates indicate that genuine and detectable genetic variation exists for the growth traits in YFC, thereby supporting the subsequent GWAS analysis.

Pearson analysis results showed that BW was significantly correlated with KL, BSL, TC, CW, and CD (*r* = 0.305, 0.517, 0.496, 0.594, 0.303, *p* < 0.01) (Fig. 5). KL was significantly correlated with BSL, TC, CW and CD (*r* = 0.43, 0.275, 0.272, 0.185, *p* < 0.01) (Fig. 5). BSL was significantly correlated with TL, TC, CW and CD (*r* = 0.346, 0.324, 0.437, 0.262, *p* < 0.01), and TL was significantly correlated with TC (*r* = 0.122, *p* < 0.05), and CW, CD (*r* = 0.208, 0.254, *p* < 0.01) (Fig. 5). In addition, TC was significantly correlated with CW and CD (*r* = 0.348, 0.261, *p* < 0.01), while CW and CD were also significantly correlated (*r* = 0.2, *p* < 0.01) (Fig. 5).

### Genome wide association study for body weight and size traits

After the quality control, genotypes for 12,644,364 high-confidence SNPs and 1,382,994 high-quality InDel on the 313 female individuals of YFC breed were retained for GWAS analyses. The QQ plots of GWAS using the SNP data (Additional file S10: Figure S4) and InDel data (Additional file 11: Figure S5) showed that *λ* values approximated to 1, indicating an effective control of confounding factors such as population stratification and relatedness in the GWAS analysis.

**Fig. 6:**
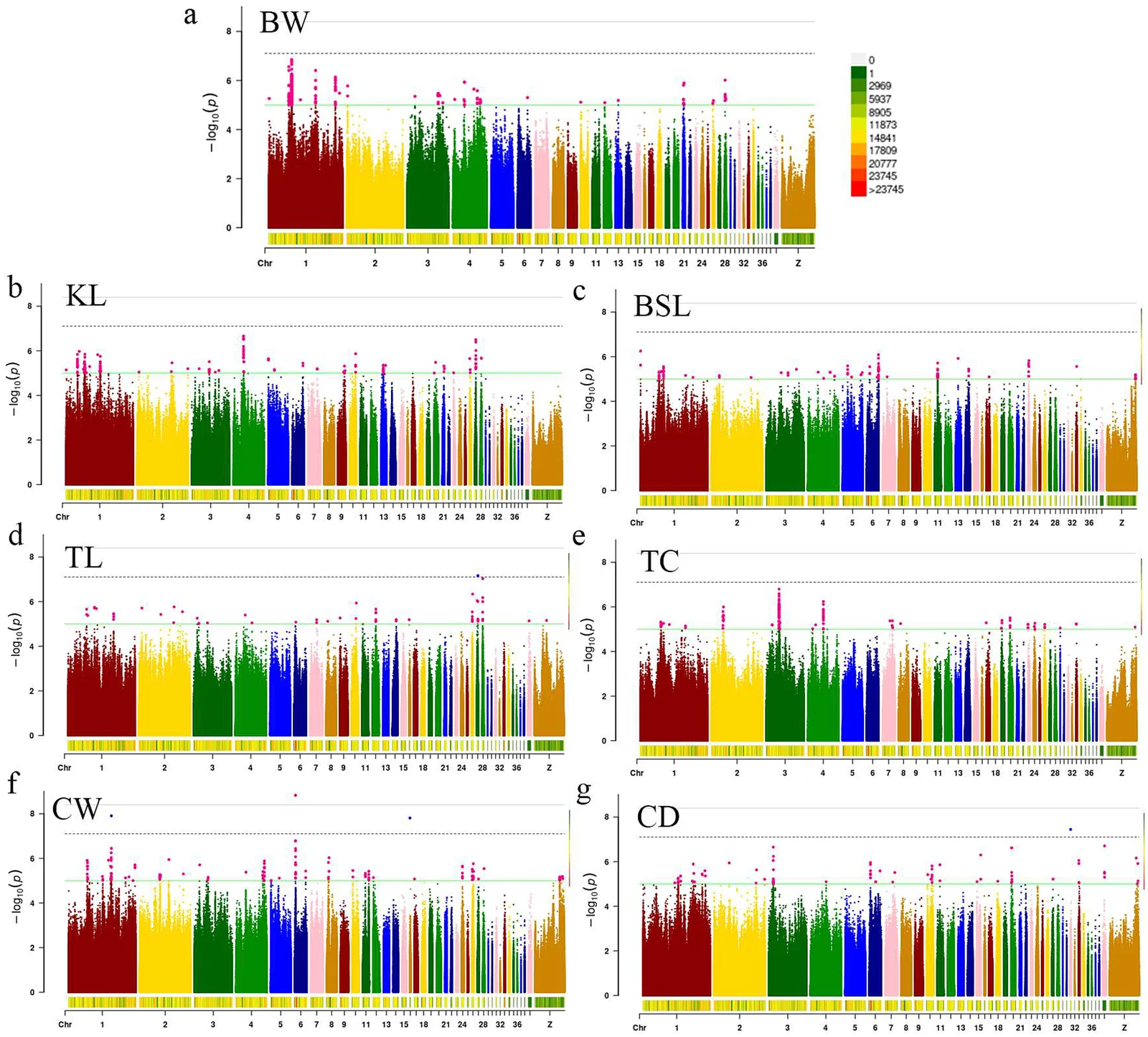
Manhattan plots of GWAS for growth traits in YFC using the SNP data. (**a**) GWAS analysis for body weight using SNP data. (**b**) GWAS analysis for keel length using SNP data. (**c**) GWAS analysis for body slope length using SNP data. (**d**) GWAS analysis for tibial length using SNP data. (**e**) GWAS analysis for tibial circumference using SNP data. (**f**) GWAS analysis for chest width using SNP data. (**g**) GWAS analysis for chest depth using SNP data. Each dot represents a SNP in the dataset. The x-axis of each plot represents the physical position of each SNP, and the y-axis displays the negative log10 of *p*-values for each SNP included in the GWAS. The suggestive significance threshold was set at -log10(*P*)=7.10, and the genome-wide significance threshold was -log10(*P*)=8.10. The green line -log10(*P*)=5 indicates an exploratory screening threshold used to prioritize putative loci for follow-up analysis

After the GWAS analysis, a total of 243 SNPs were identified for the BW, with the majority of SNPs located on chromosome 1 (60.5–60.8 Mb), primarily encompassing the *RAD52* and *ERC1* genes (Fig. 6a, Table 3 and Additional file 12: Table S7). Several other genes potentially associated with BW were also annotated, including *SYN3*, *ATP8A2*, *DVL1*, *PDGFRA*, *PPARGC1A* and *AGRN* (Fig. 6a, Table 3 and Additional file 12: Table S7). For KL, 233 SNPs were found to be associated, on chromosomes 1 (*RFX4*, *SLC41A2*, *IGF1* and *TMPRSS15*), 4 (*INPP4B*), 9 (*MECOM*), 10 (*MEGF11*), and 13 (*CCNJL*) (Fig. 6b, Table 3 and Additional file 13: Table S8). Furthermore, 117 SNPs were associated with BSL, with major annotations including the *SLC41A2*, *CHST11*, *LOC124417413*, *RBFOX1*, and *PHACTR4* genes (Fig. 6c, Table 3 and Additional file 14: Table S9). For TL, 58 SNPs were detected, with a suggestively significant SNP on chromosome 27 located within the *IGF2BP1* gene (Fig. 6d, Table 3 and Additional file 15: Table S10). We also found 136 SNPs associated with TC, predominantly located on chromosomes 2, 3, and 4, with *SCRN1*, *SUSD4*, *ERO1B*, *RAB4A*, and *MAPK10* as candidate genes (Fig. 6e, Table 3 and Additional file 16: Table S11). For CW, 151 associated SNPs were identified, with one SNP on chromosome 6 (*GRID1*) reaching the genome-wide significance, while other suggestively significant SNPs were located on chromosomes 1 (*MID1*) and 16 (*LOC121106900*) (Fig. 6f, Table 3, and Additional file 17: Table S12). For CD, 84 associated SNPs were identified, including one suggestively significant SNP on chromosome 31 (*ENSGALG00010005960* gene), as well as SNPs in *NDUFV3* on chromosomes 1 and in *SPTLC3*, *ANKEF1*, and *PAK5* on chromosomes 3 (Fig. 6g, Table 3, and Additional file 18: Table S13).

**Table 3:** A partial overview of the significant SNPs associated with growth traits in YFC

Trait	Chr	Base-pair region		nSNP	Related gene
		Star	End		
	1	53021097	60793687	155	*SYN3*, *LOC112532681*, *PWP1*, *RAD52*, *ERC1*, *LOC121107293*
		123878921	124140066	12	*FRMPD4*, *MSL3*
		176600254	176627471	16	*ATP8A2*
BW	2	2274478	2275630	2	*OBSCN*
	3	82309127	82329620	9	*SMAP1*
	4	29743091	73458572	19	*INPP4B*, *ZNF827*, *PDGFRA*, *LOC112532345*, *PPARGC1A*
	21	2297217	2298010	3	*DVL1*
	1	53697060	55381874	30	*RFX4*, *SLC41A2*, *LOC107053530*, *IGF1*
		99710626	99748741	16	*TMPRSS15*
	3	50911307	50915184	6	*ARID1B*
	4	29872363	30902842	20	*INPP4B*, *ZNF827*
KL	5	1317298	1317655	4	*LUZP2*
	9	19697181	19704669	8	*MECOM*
	10	18228739	18243007	5	*MEGF11*
	13	7478581	7489908	21	*CCNJL*
	27	3019756	3044567	9	*LOC121107680*, *NGFR*, *LOC121107681*
	1	54375186	67521238	23	*SLC41A2*, *CHST11*, *LOC124417413*
	4	65292735	65292804	3	*SCFD2*
BSL	5	54262922	54263068	2	*RTN1*
	6	9654745	9656961	3	*ANK3*
	14	10976579	10976621	3	*RBFOX1*
	23	2493022	3375265	7	*PHACTR4*, *LOC101748601*
	1	54473497	54474403	2	*CHST11*
	12	10576762	10577193	5	*TXNRD3*
TL	14	11002643	11003555	2	*RBFOX1*
	27	3268533	3331343	2	*IGF2BP1*, *LOC112530346*
	1	61039625	61041199	2	*CACNA2D4*
	2	33776116	33804788	5	*SCRN1*
TC	3	17487837	39955082	63	*SUSD4*, *ERO1B*, *RAB4A*
	4	45552118	45593971	18	*MAPK10*
	19	1167889	1168305	2	*MTMR4*
CW	1	53983912	54860353	10	*NUAK1*, *STAB2*
		123831816	124816008	19	*FRMPD4*, *MSL3*, *ARHGAP6*, *MID1*
	2	59079788	60963300	12	*CDKAL1*, *RNF144B*, *JARID2*
	4	78756034	78832047	4	*CRMP1*, *C4H4ORF50*
	6	2801611	3120294	14	*GRID1*, *LDB3*
	8	5304427	7213069	5	*PBX1*, *RABGAP1L*
	10	2476009	2477219	2	*EDC3*
	11	8017806	8018042	2	*PLEKHF1*
		18106209	18106213	3	*CDT1*
	16	216003	216003	1	*LOC121106900*
	24	2309187	2327373	5	*OPCML*
	26	926514	926671	2	*IGFN1*
		3875254	3875625	3	*SYT6*
	1	102411979	146400638	9	*LOC121109148*, *NDUFV3*, *LOC121108178*
CD	3	12560433	13476113	13	*SPTLC3*, *LOC121110388*, *ANKEF1*, *PAK5*
	31	808274	808274	1	*ENSGALG00010005960*

Further analysis through GWAS based of InDels revealed 27, 38, 11, 3, 14, 9, and 15 InDels to be associated with BW, KL, BSL, TL, TC, CW, and CD, respectively (Additional file 19: Figure S6 and Additional file 20: Table S14). Some of the genes identified in the SNP-based GWAS were also detected in the InDel-based GWAS, including *RAD52*, *ERC1*, and *ATP8A2* for BW (Additional file 19: Figure S6a, Additional file 20: Table S14, and Additional file 21: Table S15); *RFX4*, *TMPRSS15*, *INPP4B*, *LUZP2*, and *CCNJL* for KL (Additional file 19: Figure S6b, Additional file 20: Table S14, and Additional file 22: Table S16); *RBFOX1* for BSL (Additional file 19: Figure S6c, Additional file 20: Table S14, and Additional file 23: Table S17); *IGF2BP1* for TL (Additional file 19: Figure S6d, Additional file 20: Table S14, and Additional file 29: Table S18); *SCRN1*, *ERO1B*, *RAB4A*, and *MAPK10* for TC (Additional file 19: Figure S6e, Additional file 20: Table S14, and Additional file 25: Table S19); *MID1*, *GRID1*, *ARHGAP6*, and *FRMPD4* for CW (Additional file 19: Figure S6f, Additional file 20: Table S14, and Additional file 26: Table S20); and *LOC121109148* for CD (Additional file 19: Figure S6g, Additional file 20: Table S14, and Additional file 27: Table S21).

### Functional annotation of candidate genes related to growth traits in female YFCs

Gene annotation of the GWAS screened regions was performed (Table 3 and Additional file 20: Table S14), and 247 candidate genes identified from the SNP-based GWAS were subjected to GO and KEGG enrichment analysis, yielding 13 significant KEGG pathways and 256 significant GO terms (Additional file 28: Figure S7 and Additional file 29: Table S22). KEGG enrichment analysis revealed several pathways related to skeletal development and structural maintenance, including Wnt signaling (gga04310), phosphatidylinositol signaling system (gga04070), inositol phosphate metabolism (gga00562), and focal adhesion (gga04510) (Additional file 28: Figure S7a and Additional file 29: Table S22). These pathways principally encompass the following genes: *APC2*, *WNT5B*, *DVL1*, *RUVBL1*, *MAPK10*, *MTMR4*, *DGKI*, *INPP4B*, *INPP5F*, *SRC*, *PDGFRA*, *IGF1*, *PDGFD*, and *PAK5* (Additional file 29: Table S22). Pathways related to cytoskeletal dynamics and muscle development were also identified, including the MAPK signaling pathway (gga04010) and regulation of actin cytoskeleton (gga04810), which encompass key genes such as *PDGFRA*, *IGF1*, *PDGFD*, *MECOM*, *MAPK10*, *NGFR*, *CACNA2D2*, *CACNA2D4*, *APC2*, *PAK5*, and *SRC* (Additional file 28: Figure S7a and Additional file 29: Table S22).

Several GO terms related to body weight trait were enriched, such as positive regulation of multicellular organism growth (GO:0040018), positive regulation of growth hormone secretion (GO:0060124) and growth factor receptor binding (GO:0070851) (Additional file 28: Figure S7b and Additional file 29: Table S22). The following genes were enriched within these terms: *ATP8A2*, *SH3P×D2B*, *GHRH*, *ADCYAP1*, *SRC*, and *PDGFD* (Additional file 29: Table S22). Moreover, GO terms related to skeletal development were also identified, including skeletal muscle acetylcholine-gated channel clustering (GO:0071340) and positive regulation of osteoblast differentiation (GO:0045669) (Additional file 28: Figure S7b and Additional file 29: Table S22). Genes significantly enriched in these functional categories include *AGRN*, *DVL1*, *JAG1*, *SMAD1*, and *IGF1* (Additional file 29: Table S22).

## Discussion

### Background and significance of frizzled feathers in YFC

Feathers exhibited complex forms and serve as an excellent model for studying the development and evolution of morphologies. Advancements in chicken genomics research and the feather phenotypic variation among YFC and non-frizzled feather chickens have offered an excellent opportunity to investigate the genetic foundations of feather formation [41]. Given that feathers represent some of the most distinctive external traits in chickens, elucidating its underlying genetic mechanisms will markedly improve the accuracy of identifying YFC chicken breeds and populations.

YFC is a poultry breed of great research interest due to its frizzled feather morphology, which resulted from defective feather formation [8], yet it also confers certain adaptive advantages. For instance, frizzled feathers equips YFC with a loose, highly ventilated plumage that markedly improves sensible heat loss under tropical heat and humidity. In indigenous Nigerian breeds, frizzled feather roosters yielded more semen than their normal feather counterparts under tropical humid conditions [42]. And laying hens carrying the frizzle allele showed simultaneous gains in egg number and shell quality [43]. Previous research has documented potential causative genes for frizzled feathers, notably *KRT75* (present nomenclature: *KRT6A*) and *KRT75L4* [4, 13]. With the rapid development of sequencing technologies nowadays, candidate genes associated with the frizzled feather trait were identified at the whole-genome level through novel sequencing technologies in this study.

### Candidate gene mapping of the frizzled feather trait under whole-genome analysis

The application of selection signatures to identify genomic regions can provide meaningful perspectives on the influence of selection pressures on economically significant traits with adaptive importance [44, 45]. Different analytical approaches prioritized distinct aspects of the dataset, and relying solely on one approach to identify selection signatures may introduce bias [46]. In this study, three selective scanning methods (Fst, ROD, and XPCLR) were employed to identify selection signatures related to the frizzled feather trait in YFC, leading to the discovery of a substantial number of candidate genes. Through integration of the three methods, 267 overlapping genes were identified, which related to disease resistance and growth, improvement of meat quality, frizzle feather, etc. While the cross-validation among three complementary methods helps mitigate the arbitrariness of a single threshold and reduces false positives, we acknowledge that permutation-based or FDR approaches could provide additional statistical support for the candidate regions identified in this study. Future studies incorporating these correction methods would further strengthen the confidence of the identified selection signatures.

A particularly noteworthy finding was the identification of keratin family genes, which are involved in keratin filament (GO:0045095). The modification of feather type has been ascribed to variations in keratin genes [47]. It was reported that *KRT17* regulates the hair follicle cycle in mammals [48] and that *KRT74* plays a critical role in fine-wool production of Chinese sheep [49]. In addition, *KRT75* and *KRT75L4* have previously been found to be associated with the frizzled feather trait in chicken [4, 13].

In this study, the *KRT5* (keratin 5) and *KRT3* (keratin 3) genes had the strongest selection signals and also showed significant *p*-values in GWAS. The *KRT5* is expressed in the keratinocytes of the companion layer, matrix, and medulla of the mammalian hair follicle [50]. Mutations in these genes have been shown to significantly affect feather development and regeneration, including branch defects and growth arrest by expressing *KRT5* mutant forms related to human diseases in a chicken model [51]. Moreover, overexpression of *KRT5* mutation in regenerating contour feathers also led to deformities in feather structure [52]. On the other hand, *KRT3* is one of the genes specific to the structure and function of the corneal epithelium [53], but the functional implications of *KRT3* in feather development await in-depth exploration. Thus, it is reasonable to speculate that alterations in the *KRT5* and *KRT3* genes could also correlate with the frizzled feather characteristic in chickens, in addition to the acknowledged *KRT75* and *KRT75L4* genes.

### Marker validation and practical utility

To further validate the identified associations of *KRT5* and *KRT3* with frizzled feather trait, three significant loci located on *KRT5* and *KRT3* were selected. The SNPs in *KRT5* and *KRT3* effectively and clearly distinguished frizzled feather breed from normal feather breeds in our test samples. However, two individuals in the HX breed shared the same genotype as the Indel of *KRT5* in YFC, possibly due to genetic exchange resulting from the geographical proximity between HX and YFC. Although the Indel site cannot perfectly distinguish frizzled feather breed from normal feather, its detection efficiency is relatively high compared to SNP sites, thus retaining auxiliary diagnostic value. Therefore, by using these three SNPs and Indel in combination, we could accurately identify the frizzled feather chicken breed.

The 15 bp deletion in *KRT75L4*, which has been previously reported to be associated with the frizzle phenotype, was confirmed to be present in the YFC breed. In our dataset, all heterozygotes at the *KRT3* and *KRT5* loci displayed normal plumage, indistinguishable from wild-type. This strongly suggests that these SNPs are not the primary causal variants but are instead in LD with the true causal mutation (e.g., the *KRT75L4* 15bp deletion). Thus, while the *KRT3*/*KRT5* loci are significantly associated with the frizzle phenotype in the YFC breed, they are not the causative variants but rather serve as indirect genetic markers. Nonetheless, they remain practically useful for breed identification and marker-assisted selection. Importantly, by employing an integrated strategy combining selection signature analysis (Fst, ROD, XPCLR) with genome-wide association studies (GWAS), we achieved cross-validation between selective sweep regions and phenotypic associations, enhancing the reliability of these association loci.

### Heritability of growth traits

The estimated heritability of seven growth traits in YFC ranged from 0.37 (TC) to 0.75 (TL), reflecting substantial genetic contributions to phenotypic variation. The heritability estimates for body weight (0.47) in this study were generally consistent with previous reports, such as for body weight (0.44) in broiler chickens [54] and for body weight (0.47) in Korean Native Chickens [55]. Heritabilities for other growth traits, including KL (0.64), BSL (0.54), TL (0.75), CW (0.56), and CD (0.59), were comparable to findings from earlier investigations [2] and were classified as high-heritability traits. In contrast, TC, which had the lowest heritability (0.37), was comparable to the heritability of 0.33 reported for 18-week-old TC in Wenshang Barred chickens [2].

### GWAS significance and threshold selection

Genome-wide studies on body weight and body size traits of the YFC breed are limited, which prompted us to conduct relevant investigations. The mixed linear model (MLM) approach has been widely used in GWAS because it can effectively control the false positive rate of SNP or InDel detection. After Bonferroni correction, very few loci reached genome-wide significance. Specifically, only one genome-wide significant locus was identified for CW, located on chromosome 6, with *GRID1* annotated as the candidate gene. *GRID1* has been reported to be associated with body size traits and it is one of the functional candidate genes for sheep body size [56]. Suggestively significant loci were found only for TL, CW, and CD. One was detected for TL on chromosome 27 (annotated as *IGF2BP1*). It was reported that *IGF2BP1* was associated with shark length trait [2], which is consistent with the findings of our study. And two genes were detected for breast width, on chromosomes 1 (annotated as *MID1*) and 16 (annotated as *LOC121106900*). However, none of these genes have been previously reported to be associated with body size. One genomic region was detected for breast depth on chromosome 28, which had no annotated gene. The limited sample size, combined with the fact that body weight and body size are complex quantitative traits governed by many small-effect loci, resulted in very few loci reaching significance following the stringent Bonferroni correction.

Although Bonferroni correction is a common method in GWAS models, it was overly stringent for the growth trait data, leading to the loss of many important small effect loci [57]. Therefore, we adopted a new threshold (–log10(*p*) = 5) to capture additional candidate genes [39] to make full use of the current data to provide valuable clues for subsequent research. The following are discussions of candidate genes that were identified based on the new threshold.

### Exploratory analysis of potential candidate genes for growth and body-size traits

*ERC1*, *RAD52*, *ATP8A2*, and *PPARGC1A* were identified to be associated with body weight through GWAS analysis. It has been reported that the ERC1 protein of the RIM-binding protein family could participate in regulation of neurotransmitter release [58]. A hemizygous deletions of *ERC1* may be involved in the development of neurological disorders, leading to developmental delay [59]. Furthermore, *ERC1* has been found to be significantly associated with body weight in Chengkou Mountain Chicken [60]. The *ATP8A2* gene has been identified as a candidate gene for postweaning body weight traits in Lori-Bakhtiari sheep [61]. In addition, *PPARGC1A* was identified a potential gene associated with body weight in Korean native chickens [55]. Therefore, *ERC1*, *RAD52*, *ATP8A2*, and *PPARGC1A* are likely candidate genetic markers for body weight traits in chicken breeding.

We recognized *RFX4*, *TMPRSS15*, *INPP4B*, *LUZP2*, and *CCNJL* had genetic variants (SNPs and Indels) that showed significant associations with keel length (KL). The *RFX4* gene may serve as an important candidate gene for reproductive and behavioral traits in chickens [62], but its potential role in body size regulation remains unreported. The *TMPRSS15* gene was proposed as a strong candidate gene for body size traits in Yorkshire pigs [63]. The *INPP4B* gene was associated with bone mineral density variability in humans and a deficient *INPP4B* increased the possibility of bone mass loss and osteoporosis in vivo mice [64]. The *LUZP2* gene had been identified as a candidate gene associated with body parameters in sheep [65], while *CCNJL* gene has not been functionally described yet in chickens or other birds. The *IGF1* gene, which was only identified by SNP GWAS for KL trait also showed associations with body size traits. *IGF1* promotes skeletal growth by exerting insulin-like actions that enlarge chondrocytes, thereby regulating development and body size [66]. For both body slope length (BSL), only one common gene *RBFOX1* was identified by both SNP and InDel GWAS. *RBFOX1*, an RNA-binding protein, regulates skeletal muscle alternative splicing and RNA processing, and plays a critical role in maintaining cardiac function by modulating calcium ion related gene expression [67]. To our knowledge, no studies have yet reported its association with body size traits.

For the tibial circumference, genes such as *SCRN1*, *ERO1B*, *RAB4A*, and *MAPK10* were recognized as candidate genes. *SCRN1*, *ERO1B*, and *RAB4A* have not been found to be associated with body size traits but they have been studied and reported in relation to the fertility of mouse sperm [68], redox steady state in beef cattle [69], and litter size in sheep [70], respectively. The *MAPK10* gene was associated with growth hormone synthesis and secretion in Chinese Holstein Cattle and shows significant correlations with body weight and height in Nanyang Cattle [71, 72]. The *ARHGAP6* and *FRMPD4* genes were detected as candidate genes associated with chest width but, to the best of our knowledge, no published studies have documented an association between these genes and morphological traits related to body size.

A caveat of the present study is the lack of effect size estimates (e.g., β and PVE) for the associated variants, as these statistics were not retained in the initial GWAS data extraction. While this prevents a quantitative assessment of the phenotypic contribution of each locus, we have instead focused on functional annotation and cross-referencing with published studies to substantiate the candidacy of these regions. We acknowledge that future replication studies in expanded populations are necessary to precisely estimate the genetic effects and validate the robustness of these associations.

## Conclusions

In order to investigate the genomic structure and genetic determinants of frizzled feather, we conducted the first genome wide analysis in YFC for frizzled feather traits using whole genome resequencing data. Integration of selection signature analysis with GWAS pointed to *KRT5* and *KRT3* as two potential candidate genes associated with the frizzle phenotype. Three specific variants in *KRT5* and *KRT3* genes effectively distinguished frizzled feather from non-frizzled phenotypes, serving as new genetic markers. We also identified candidate genes potentially associated with growth traits through GWAS, including *ERC1*, *RAD52*, *ATP8A2*, *PPARGC1A*, *RFX4*, *TMPRSS15*, *INPP4B*, *LUZP2*, *CCNJL*, *IGF1*, *RBFOX1*, *IGF2BP1*, *SCRN1*, *ERO1B*, *RAB4A*, *MAPK10*, *MID1*, *GRID1*, *ARHGAP6*, *FRMPD4*, and *LOC121109148*. In summary, our findings contribute to a better understanding of the genetic basis of frizzled feather and growth traits in YFC, which provide valuable insights into breeding under hot and humid climate.

## Electronic supplementary material

Below is the link to the electronic supplementary material.

Supplementary material 1: Sample information and geographic distribution of the chickens included in this study. Description: Breed-level sample information, including breed names and sample sizes, sampling locations, feather phenotypes, and experimental purposes, together with representative breed photographs and their corresponding geographic locations on a map of China

Supplementary material 2: QQ plots of GWAS for frizzle feather trait. Description: Quantile-quantile plots comparing observed and expected−log10(P) values from the GWAS of the frizzle-feather trait for SNP and InDel variants; the red dashed lines indicate the expected nulldistributions.

Supplementary material 3: Sanger sequencing results for the 15 bp deletion locus in the *KRT75L4* gene using samples from the present study. YFC: Yuexi Frizzled Feather Chicken; HC: Huaixiang Chicken. Description: Representative Sanger sequencing chromatograms from one YFC and four HC samples validating the 15-bp deletion in *KRT75L4*. The deleted segment is highlighted; the YFC sample carries the deletion, whereas the HC samples show the corresponding intact sequence.

Supplementary material 4: QQ plots of GWAS for growth traits based on SNP data. The λ value is the genomic inflation factor, which quantifies the overall deviation between the observed P-value distribution and the expected distribution. The λ value close to 1 indicates that the results are in their ideal state. BW: body weight, KL: keel length, BSL: body slope length, TL: tibial length, TC: tibial circumference, CW: chest width, CD: chest depth. Description: Seven QQ plots comparing observed and expected −log10(P) values from SNP-based GWAS of body weight and six body-size traits. Each panel reports the genomic inflation factor (λ), with the red dashed lines indicating the expected null distributions and shaded areas indicating confidence intervals.

Supplementary material 5: QQ plots of GWAS for growth traits based on InDel data. The λ value is the genomic inflation factor, which quantifies the overall deviation between the observed P-value distribution and the expected distribution. The λ value close to 1 indicates that the results are in their ideal state. BW: body weight, KL: keel length, BSL: body slope length, TL: tibial length, TC: tibial circumference, CW: chest width, CD: chest depth. Description: Seven QQ plots comparing observed and expected −log10(*P*) values from InDel-based GWAS of body weight and six body-size traits. Each panel reports the genomic inflation factor (λ), with red dashed lines indicating the expected null distributions and shaded areas indicating confidence intervals.

Supplementary material 6: Manhattan plots of GWAS for growth traits in YFC using the InDel data. (a) GWAS analysis for body weight using InDel data. (b) GWAS analysis for keel length using InDel data. (c) GWAS analysis for body slope length using InDel data. (d) GWAS analysis for tibial length using InDel data. (e) GWAS analysis for tibial circumference using InDel data. (f) GWAS analysis for chest width using InDel data. (g) GWAS analysis for chest depth using InDel data. Each dot represents an InDel in the dataset. The x-axis of each plot represents the physical position of each InDel, and the y-axis displays the negative log10 of p-values for each InDel included in the GWAS. The suggestive significance threshold was set at -log10(*P*)=6.14, and the genome-wide significance threshold was -log10(*P*)=7.44. The green line -log10(*P*)=5 indicates an exploratory screening threshold used to prioritize putative loci for follow-up analysis. Description: Seven Manhattan plots (a–g) showing InDel-based GWAS results for body weight, keel length, body slope length, tibial length, tibial circumference, chest width, and chest depth in YFC chickens. Each point represents an InDel plotted by genomic position against −log10(*P*).

Supplementary material 7: Functional enrichment analysis of candidate genes associated with growth traits. (a) Bubble chart illustrating the Kyoto Encyclopedia of Genes and Genomes (KEGG) pathway analysis results. (b) Bubble chart illustrating the Gene Ontology (GO) enrichment analysis results. Description: Bubble charts showing the functional enrichment of candidate genes associated with growth traits, including KEGG pathway results (a) and Gene Ontology results (b). Point size indicates gene count, color indicates the P value, and the x-axis indicates the gene ratio.

Supplementary material 8: Sample resequencing information for the 356 individuals of YFC chicken. Description: Per-sample resequencing information for YFC chickens, including sample identifiers, sex, feather color, clean read and base counts, read length, Q20 and Q30 quality percentages, GC content, and sequencing depth.

Supplementary material 9: Sample resequencing information for the 60 individuals of QC chicken. Description: Per-sample resequencing information for 60 QC chickens, including sample identifiers, sex, clean read and base counts, read length, GC content, Q30 percentage, and sequencing depth.

Supplementary material 10: Sample information for the 25 individuals of QXC chicken. Description: Run-level sequencing metadata for 25 QXC chicken samples, including SRA run and experiment accessions, assay type, breed, number of sequenced bases, sequencing platform and instrument, library source and layout, and sex.

Supplementary material 11: Primer of *KRT5*, *KRT3* and *KRT75L4* for PCR. Description: PCR primer pairs for *KRT5*, *KRT3*, and *KRT75L4*, including primer names, forward and reverse primer sequences, annealing temperatures, and expected PCR product lengths.

Supplementary material 12: Overlapping regions and genes of plumage type were filtered out by using three selection methods. Description: Candidate genomic regions and associated genes identified for plumage type using three selection-scan methods (FST, ROD, and XPCLR) at the top 5% threshold, together with the overlapping regions and genes shared among these methods.

Supplementary material 13: KEGG and GO functional enrichment analysis of candidate genes for the frizzle-feather trait from the selective sweep. Description: KEGG pathway and Gene Ontology enrichment results for candidate genes identified through selective-sweep analysis of the frizzle-feather trait, including enriched terms, database identifiers, input and background gene counts, raw and corrected *P* values, and the mapped candidate genes.

Supplementary material 14: Significant SNPs associated with Body Weight in YFC by GWAS. Description: GWAS results for body weight in YFC chickens, listing 243 significant SNPs with their identifiers, chromosome and base-pair positions, *P* values, and mapped gene annotations.

Supplementary material 15: Significant SNPs associated with Keel Length in YFC by GWAS. Description: GWAS results for keel length in YFC chickens, listing 233 significant SNPs with their identifiers, chromosome and base-pair positions, *P* values, and mapped gene annotations where available.

Supplementary material 16: Significant SNPs associated with Body Slope Length in YFC by GWAS. Description: GWAS results for body slope length in YFC chickens, listing 117 significant SNPs with their identifiers, chromosome and base-pair positions, P values, and mapped gene annotations where available.

Supplementary material 17: Significant SNPs associated with Tibial Length in YFC by GWAS. Description: GWAS results for tibial length in YFC chickens, listing 58 significant SNPs with their identifiers, chromosome and base-pair positions, P values, and mapped gene annotations where available.

Supplementary material 18: Significant SNPs associated with Tibial Circumference in YFC by GWAS. Description: GWAS results for tibial circumference in YFC chickens, listing 136 significant SNPs with their identifiers, chromosome and base-pair positions, P values, and mapped gene annotations where available.

Supplementary material 19: Significant SNPs associated with Chest Width in YFC by GWAS. Description: GWAS results for chest width in YFC chickens, listing 151 significant SNPs with their identifiers, chromosome and base-pair positions, P values, and mapped gene annotations where available.

Supplementary material 20: Significant SNPs associated with Chest Depth in YFC by GWAS. Description: GWAS results for chest depth in YFC chickens, listing 83 significant SNPs with their identifiers, chromosome and base-pair positions, P values, and mapped gene annotations where available.

Supplementary material 21: A partial overview of the significant InDels associated with growth traits in YFC. Description: Selected genomic regions containing significant growth-trait-associated InDels in YFC chickens, summarized by trait, chromosome and base-pair coordinates, number of InDels, and related genes.

Supplementary material 22: Significant InDels associated with Body Weight in YFC by GWAS. Description: GWAS results for body weight in YFC chickens, listing 27 significant InDels with their identifiers, chromosome and base-pair positions, P values, and mapped gene annotations where available.

Supplementary material 23: Significant InDels associated with Keel Length in YFC by GWAS. Description: GWAS results for keel length in YFC chickens, listing 38 significant InDels with their identifiers, chromosome and base-pair positions, P values, and mapped gene annotations where available.

Supplementary material 24: Significant InDels associated with Body Slope Length in YFC by GWAS. Description: GWAS results for body slope length in YFC chickens, listing 11 significant InDels with their identifiers, chromosome and base-pair positions, P values, and mapped gene annotations where available.

Supplementary material 25: Significant InDels associated with Tibial Circumference in YFC by GWAS. Description: GWAS results for tibial circumference in YFC chickens, listing 14 significant InDels with their identifiers, chromosome and base-pair positions, *P* values, and mapped gene annotations where available.

Supplementary material 26: Significant InDels associated with Chest Width in YFC by GWAS. Description: GWAS results for chest width in YFC chickens, listing nine significant InDels with their identifiers, chromosome and base-pair positions, *P* values, and mapped gene annotations where available.

Supplementary material 27: Significant InDels associated with Chest Depth in YFC by GWAS. Description: GWAS results for chest depth in YFC chickens, listing 15 significant InDels with their identifiers, chromosome and base-pair positions, *P* values, and mapped gene annotations where available.

Supplementary material 28: KEGG and GO functional enrichment analysis of candidate genes from body weight and body size traits by GWAS. Description: KEGG pathway and Gene Ontology enrichment results for GWAS candidate genes associated with body weight and body-size traits, including enriched terms, database identifiers, input and background gene counts, P values, and mapped candidate genes.

Supplementary material 29: Table S18. Format: .xlsx Title: Significant InDels associated with Tibial Length in YFC by GWAS. Description: GWAS results for tibial length in YFC chickens, listing three significant InDels with their identifiers, chromosome and base-pair positions, P values, and mapped gene annotations where available.

## Data Availability

The sequence data used in this study has been submitted to National Center for Biotechnology in formation (NCBI) with accession number PRJNA1303340.

## References

[1] Li D, Li Y, Li M, Che T, Tian S, Chen B, et al. Population genomics identifies patterns of genetic diversity and selection in chicken. BMC Genomics. 2019;20(1):263. 10.1186/s12864-019-5622-4. PMC6446315 30940068

[2] Wang J, Liu J, Lei Q, Liu Z, Han H, Zhang S, et al. Elucidation of the genetic determination of body weight and size in Chinese local chicken breeds by large-scale genomic analyses. BMC Genomics. 2024;25(1):296. 10.1186/s12864-024-10185-6. PMC10956266 38509464

[3] Chang WL, Wu H, Chiu YK, Wang S, Jiang TX, Luo ZL, et al. The making of a flight feather: bio-architectural principles and adaptation. Cell. 2019;179(6):1409–23.e17. 10.1016/j.cell.2019.11.008. PMC6953487 31778655

[4] Ng CS, Wu P, Foley J, Foley A, McDonald ML, Juan WT, et al. The chicken frizzle feather is due to an α-keratin (KRT75) mutation that causes a defective rachis. PLoS Genet. 2012;8(7):e1002748. 10.1371/journal.pgen.1002748. PMC3400578 22829773

[5] Wu H, Chuang TC, Liao WC, Chi KJ, Ng CS, Cheng HC, et al. Modification of keratin integrations and the associated morphogenesis in frizzling chicken feathers. Biol (Basel). 2024;13(7):464. 10.3390/biology13070464. PMC11273737 39056659

[6] Li J, Wang Z, Lubritz D, Arango J, Fulton J, Settar P, et al. Genome-wide association studies for egg quality traits in White Leghorn layers using low-pass sequencing and SNP chip data. J Anim Breed Genet. 2022;139(4):380–97. 10.1111/jbg.12679. 35404478

[7] Feng C, Gao Y, Dorshorst B, Song C, Gu X, Li Q, et al. A cis-regulatory mutation of PDSS2 causes silky-feather in chickens. PLoS Genet. 2014;10(8):e1004576. 10.1371/journal.pgen.1004576. PMC4148213 25166907

[8] Chen B, Xi S, El-Senousey HK, Zhou M, Cheng D, Chen K, et al. Deletion in KRT75L4 linked to frizzle feather in Xiushui Yellow chickens. Anim Genet. 2022;53(1):101–07. 10.1111/age.13158. 34904261

[9] Fathi MM, Galal A, Radwan LM, Abou-Emera OK, Al-Homidan IH. Using major genes to mitigate the deleterious effects of heat stress in poultry: an updated review. Poult Sci. 2022;101(11):102157. 10.1016/j.psj.2022.102157. PMC9513277 36167017

[10] Kpomasse CC, Oke OE, Houndonougbo FM, Tona K. Broiler production challenges in the tropics: a review. Vet Med Sci. 2021;7(3):831–42. 10.1002/vms3.435. PMC8136938 33559980

[11] Liu L, Ren M, Ren K, Jin Y, Yan M. Heat stress impacts on broiler performance: a systematic review and meta-analysis. Poult Sci. 2020;99(11):6205–11. 10.1016/j.psj.2020.08.019. PMC7647856 33142538

[12] Chen CF, Foley J, Tang PC, Li A, Jiang TX, Wu P, et al. Development, regeneration, and evolution of feathers. Annu Rev Anim Biosci. 2015;3(1):169–95. 10.1146/annurev-animal-022513-114127. PMC5662002 25387232

[13] Dong J, He C, Wang Z, Li Y, Li S, Tao L, et al. A novel deletion in KRT75L4 mediates the frizzle trait in a Chinese indigenous chicken. Genet Sel Evol. 2018;50(1):68. 10.1186/s12711-018-0441-7. PMC6302451 30572816

[14] Yu Y, Hilsdorf AWS, Zhou L, Lin Q, Gao ZX. Editorial: genetics and molecular breeding in aquaculture animals. Front Genet. 2022;13:1071303. 10.3389/fgene.2022.1071303. PMC9686421 36437943

[15] Xu D, Zhu W, Wu Y, Wei S, Shu G, Tian Y, et al. Whole-genome sequencing revealed genetic diversity, structure and patterns of selection in Guizhou indigenous chickens. BMC Genomics. 2023;24(1):570. 10.1186/s12864-023-09621-w. PMC10521574 37749517

[16] Zhang X, Yang F, Zhu T, Zhao X, Zhang J, Wen J, et al. Whole genome resequencing reveals genomic regions related to red plumage in ducks. Poult Sci. 2024;103(6):103694. 10.1016/j.psj.2024.103694. PMC11068611 38663207

[17] Lyu S, Arends D, Nassar MK, Weigend A, Weigend S, Wang E, et al. High-density genotyping reveals candidate genomic regions for chicken body size in breeds of Asian origin. Poult Sci. 2023;102(1):102303. 10.1016/j.psj.2022.102303. PMC9706647 36436378

[18] Zhang Y, Wang Y, Li Y, Wu J, Wang X, Bian C, et al. Genome-wide association study reveals the genetic determinism of growth traits in a gushi-anka F2 chicken population. Heredity (Edinb). 2021;126(2):293–307. 10.1038/s41437-020-00365-x. PMC8026619 32989280

[19] Pan R, Qi L, Xu Z, Zhang D, Nie Q, Zhang X, et al. Weighted single-step GWAS identified candidate genes associated with carcass traits in a Chinese yellow-feathered chicken population. Poult Sci. 2024;103(2):103341. 10.1016/j.psj.2023.103341. PMC10776626 38134459

[20] Wang S, Wang Y, Li Y, Xiao F, Guo H, Gao H, et al. Genome-wide association study and selective sweep analysis reveal the genetic architecture of body weights in a chicken F2 resource population. Front Vet Sci. 2022;9:875454. 10.3389/fvets.2022.875454. PMC9361851 35958311

[21] Gao Y, Feng CG, Song C, Du ZQ, Deng XM, Li N, et al. Mapping quantitative trait loci affecting chicken body size traits via genome scanning. Anim Genet. 2011;42(6):670–74. 10.1111/j.1365-2052.2011.02193.x. 22035012

[22] Xie X, Shi L, Hou G, Zhong Z, Wang Z, Pan D, et al. Genome wide detection of CNV and their association with body size in Danzhou chickens. Poult Sci. 2024;103(12):104266. 10.1016/j.psj.2024.104266. PMC11426044 39293262

[23] Li Z, Nong Y, Liu Y, Wang Z, Wang J, Li Z. Genome-wide association study of body size traits in Luning chickens using whole-genome sequencing. Anim (Basel). 2025;15(7):972. 10.3390/ani15070972. PMC11987916 40218365

[24] Wu C, Dong L, Gan X, Gan F, Xu W, Lu L. Genome-wide association studies and haplotype sharing analysis targeting the growth traits in Yandang partridge chickens. Anim Biotechnol. 2023;34(6):1943–49. 10.1080/10495398.2022.2059491. 35400313

[25] Peng S, Zhang L, Ali S, Lin S, Liao Z, Peng Q, et al. Whole genome resequencing uncovers candidate genes related to plumage color in Yuexi frizzled feather chicken. Poult Sci. 2025;104(11):105680. 10.1016/j.psj.2025.105680. PMC12396410 40840286

[26] Chen Y, Chen Y, Shi C, Huang Z, Zhang Y, Li S, et al. Soapnuke: a MapReduce acceleration-supported software for integrated quality control and preprocessing of high-throughput sequencing data. GigaScience. 2018;7(1):gix120. 10.1093/gigascience/gix120. PMC5788068 29220494

[27] Li H, Durbin R. Fast and accurate short read alignment with Burrows–Wheeler transform. Bioinformatics. 2009;25(14):1754–60. 10.1093/bioinformatics/btp324. PMC2705234 19451168

[28] Li H, Handsaker B, Wysoker A, Fennell T, Ruan J, Homer N, et al. The sequence alignment/map format and SAMtools. Bioinformatics. 2009;25(16):2078–79. 10.1093/bioinformatics/btp352. PMC2723002 19505943

[29] McKenna A, Hanna M, Banks E, Sivachenko A, Cibulskis K, Kernytsky A, et al. The genome analysis toolkit: a MapReduce framework for analyzing next-generation DNA sequencing data. Genome Res. 2010;20(9):1297–303. 10.1101/gr.107524.110. PMC2928508 20644199

[30] Danecek P, Auton A, Abecasis G, Albers CA, Banks E, DePristo MA, et al. The variant call format and VCFtools. Bioinformatics. 2011;27(15):2156–58. 10.1093/bioinformatics/btr330. PMC3137218 21653522

[31] Zhang C, Dong SS, Xu JY, He WM, Yang TL. PopLDdecay: a fast and effective tool for linkage disequilibrium decay analysis based on variant call format files. Bioinformatics. 2019;35(10):1786–88. 10.1093/bioinformatics/bty875. 30321304

[32] Purcell S, Neale B, Todd-Brown K, Thomas L, Ferreira MAR, Bender D, et al. PLINK: a tool set for whole-genome association and population-based linkage analyses. Am J Hum Genet. 2007;81(3):559–75. 10.1086/519795. PMC1950838 17701901

[33] Xu L, He W, Tai S, Huang X, Qin M, Liao X, et al. VCF2Dis: an ultra-fast and efficient tool to calculate pairwise genetic distance and construct population phylogeny from VCF files. GigaScience. 2025;14:giaf032. 10.1093/gigascience/giaf032. PMC11970368 40184433

[34] Letunic I, Bork P. Interactive tree of life (iTOL) v4: recent updates and new developments. Nucleic Acids Res. 2019;47(W1):W256–9. 10.1093/nar/gkz239. PMC6602468 30931475

[35] Bhatia G, Patterson N, Sankararaman S, Price AL. Estimating and interpreting FST: the impact of rare variants. Genome Res. 2013;23(9):1514–21. 10.1101/gr.154831.113. PMC3759727 23861382

[36] Xu X, Liu X, Ge S, Jensen JD, Hu F, Li X, et al. Resequencing 50 accessions of cultivated and wild rice yields markers for identifying agronomically important genes. Nat Biotechnol. 2012;30(1):105–11. 10.1038/nbt.2050. 22158310

[37] Chen H, Patterson N, Reich D. Population differentiation as a test for selective sweeps. Genome Res. 2010;20(3):393–402. 10.1101/gr.100545.109. PMC2840981 20086244

[38] Kang HM, Sul JH, Service SK, Zaitlen NA, Kong SY, Freimer NB, et al. Variance component model to account for sample structure in genome-wide association studies. Nat Genet. 2010;42(4):348–54. 10.1038/ng.548. PMC3092069 20208533

[39] Zhang Y, Jia C, Li S, Wang S, He Z, Wu G, et al. Comparative genome-wide association study on body weight in Chinese native ducks using four models. Poult Sci. 2024;103(8):103899. 10.1016/j.psj.2024.103899. PMC11253684 38909509

[40] Ng CS, Li WH. Genetic and molecular basis of feather diversity in birds. Genome Biol Evol. 2018;10(10):2572–86. 10.1093/gbe/evy180. PMC6171735 30169786

[41] Mou C, Pitel F, Gourichon D, Vignoles F, Tzika A, Tato P, et al. Cryptic patterning of avian skin confers a developmental facility for loss of neck feathering. PLoS Biol. 2011;9(3):e1001028. 10.1371/journal.pbio.1001028. PMC3057954 21423653

[42] Machebe NS, Ezekwe AG. Ejaculate characteristics of three genotypes of local cocks in the humid tropics. Agro-Science. 2007;3(2):33. 10.4314/as.v3i2.1510.

[43] Adomako K, Olympio S, Hagan K, Hamidu JA. Effect of the frizzle gene (F) on egg production and egg quality of laying hens kept in tropical villages. Br Poult Sci. 2014;55(6):709–14. 10.1080/00071668.2014.963026. 25277785

[44] Kemper KE, Saxton SJ, Bolormaa S, Hayes BJ, Goddard ME. Selection for complex traits leaves little or no classic signatures of selection. BMC Genomics. 2014;15(1):246. 10.1186/1471-2164-15-246. PMC3986643 24678841

[45] Zhao F, McParland S, Kearney F, Du L, Berry DP. Detection of selection signatures in dairy and beef cattle using high-density genomic information. Genet Sel Evol. 2015;47(1):49. 10.1186/s12711-015-0127-3. PMC4472243 26089079

[46] Zhong ZQ, Li R, Wang Z, Tian SS, Xie XF, Wang ZY, et al. Genome-wide scans for selection signatures in indigenous pigs revealed candidate genes relating to heat tolerance. Animal. 2023;17(7):100882. 10.1016/j.animal.2023.100882. 37406393

[47] Prum RO, Williamson S. Theory of the growth and evolution of feather shape. J Exp Zool. 2001;291(1):30–57. 10.1002/jez.4. 11335915

[48] Tong X, Coulombe PA. Keratin 17 modulates hair follicle cycling in a TNFα-dependent fashion. Genes Dev. 2006;20(10):1353–64. 10.1101/gad.1387406. PMC1472909 16702408

[49] Liang B, Bai T, Zhao Y, Han J, He X, Pu Y, et al. Two mutations at KRT74 and EDAR synergistically drive the fine-wool production in Chinese sheep. J Adv Res. 2024;57:1–13. 10.1016/j.jare.2023.04.012. PMC10918353 37137429

[50] Winter H, Schissel D, Parry DAD, Smith TA, Liovic M, Birgitte Lane EB, et al. An unusual Ala12Thr polymorphism in the 1A α-helical segment of the companion layer-specific keratin K6hf: evidence for a risk factor in the etiology of the common hair disorder pseudofolliculitis barbae. J Invest Dermatol. 2004;122(3):652–57. 10.1111/j.0022-202X.2004.22309.x. 15086549

[51] Ng CS, Wu P, Fan WL, Yan J, Chen CK, Lai YT, et al. Genomic organization, transcriptomic analysis, and functional characterization of avian α- and β-keratins in diverse feather forms. Genome Biol Evol. 2014;6(9):2258–73. 10.1093/gbe/evu181. PMC4202321 25152353

[52] Ye S, Yuan X, Lin X, Gao N, Luo Y, Chen Z, et al. Imputation from SNP chip to sequence: a case study in a Chinese indigenous chicken population. J Anim Sci Biotechnol. 2018;9(1):30. 10.1186/s40104-018-0241-5. PMC5861640 29581880

[53] Sasamoto Y, Hayashi R, Park SJ, Saito-Adachi M, Suzuki Y, Kawasaki S, et al. PAX6 isoforms, along with reprogramming factors, differentially regulate the induction of cornea-specific genes. Sci Rep. 2016;6(1):20807. 10.1038/srep20807. PMC4761963 26899008

[54] Dadousis C, Somavilla A, Ilska JJ, Johnsson M, Batista L, Mellanby RJ, et al. A genome-wide association analysis for body weight at 35 days measured on 137, 343 broiler chickens. Genet Sel Evol. 2021;53(1):70. 10.1186/s12711-021-00663-w. PMC8424881 34496773

[55] Cha J, Choo H, Srikanth K, Lee SH, Son JW, Park MR, et al. Genome-wide association study identifies 12 loci associated with body weight at age 8 weeks in Korean native chickens. Genes (Basel). 2021;12(8):1170. 10.3390/genes12081170. PMC8394794 34440344

[56] Kominakis A, Hager-Theodorides AL, Zoidis E, Saridaki A, Antonakos G, Tsiamis G. Combined GWAS and ‘guilt by association’-based prioritization analysis identifies functional candidate genes for body size in sheep. Genet Sel Evol. 2017;49(1):41. 10.1186/s12711-017-0316-3. PMC5408376 28454565

[57] Dou D, Shen L, Zhou J, Cao Z, Luan P, Li Y, et al. Genome-wide association studies for growth traits in broilers. BMC Genom Data. 2022;23(1):1. 10.1186/s12863-021-01017-7. PMC8725492 34979907

[58] Held RG, Kaeser PS. ELKS active zone proteins as multitasking scaffolds for secretion. Open Biol. 2018;8(2):170258. 10.1098/rsob.170258. PMC5830537 29491150

[59] Abdelmoity AT, Hall JJ, Bittel DC, Yu S. 1.39 mb inherited interstitial deletion in 12p13.33 associated with developmental delay. Eur J Med Genet. 2011;54(2):198–203. 10.1016/j.ejmg.2010.11.010. 21144913

[60] Liu L, Wang Y, Huang Y, Wang Z, Wang Q, Wang H. Genetic diversity analysis and identification of candidate genes for growth traits in Chengkou Mountain Chicken. Int J Mol Sci. 2024;25(23):12939. 10.3390/ijms252312939. PMC11641596 39684650

[61] Almasi M, Zamani P, Mirhoseini SZ, Moradi MH. Genome-wide association study for postweaning weight traits in Lori-Bakhtiari sheep. Trop Anim Health Prod. 2021;53(1):163. 10.1007/s11250-021-02595-5. 33586021

[62] Romanov MN, Shakhin AV, Abdelmanova AS, Volkova NA, Efimov DN, Fisinin VI, et al. Dissecting selective signatures and candidate genes in grandparent lines subject to high selection pressure for broiler production and in a local Russian chicken breed of Ushanka. Genes (Basel). 2024;15(4):524. 10.3390/genes15040524. PMC11050503 38674458

[63] Liu H, Song H, Jiang Y, Jiang Y, Zhang F, Liu Y, et al. A single-step genome wide association study on body size traits using imputation-based whole-genome sequence data in Yorkshire pigs. Front Genet. 2021;12:629049. 10.3389/fgene.2021.629049. PMC8283822 34276758

[64] Ferron M, Boudiffa M, Arsenault M, Rached M, Pata M, Giroux S, et al. Inositol polyphosphate 4-phosphatase B as a regulator of bone mass in mice and humans. Cell Metab. 2011;14(4):466–77. 10.1016/j.cmet.2011.08.013. PMC3204353 21982707

[65] Krivoruchko A, Likhovid A, Kanibolotskaya A, Saprikina T, Safaryan E, Yatsyk O. Genome-wide search for associations with meat production parameters in Karachaevsky sheep breed using the Illumina BeadChip 600K. Genes (Basel). 2023;14(6):1288. 10.3390/genes14061288. PMC10298605 37372468

[66] Yan S, Gao C, Tian K, Xiao C, Shi J, Jia X, et al. Comparative population genomics analysis for chicken body sizes using genome-wide single nucleotide polymorphisms. Anim Biosci. 2025;38(4):600–11. 10.5713/ab.24.0347. PMC11917417 39482999

[67] Umar S, Zhu W, Souza-Neto F, Bender I, Wu SC, Healy CL, et al. RBFOX1 regulates calcium signaling and enhances SERCA2 translation. Cells. 2025;14(9):664. 10.3390/cells14090664. PMC12072054 40358188

[68] Houston BJ, Nagirnaja L, Merriner DJ, O’connor AE, Okuda H, Omurtag K, et al. The Sertoli cell expressed gene secernin-1 (Scrn1) is dispensable for male fertility in the mouse. Dev Dyn. 2021;250(7):922–31. 10.1002/dvdy.299. PMC9012169 33442887

[69] Polizel GHG, Santos MEPD, Cesar ASM, Diniz WJS, Ramírez-Zamudio GD, Fantinato-Neto P, et al. Late gestation maternal nutrition has a stronger impact on offspring liver transcriptome than full-gestation supplementation in beef cattle. Vet Sci. 2025;12(5):406. 10.3390/vetsci12050406. PMC12115941 40431499

[70] Esmaeili-Fard SM, Gholizadeh M, Hafezian SH, Abdollahi-Arpanahi R. Genome-wide association study and pathway analysis identify NTRK2 as a novel candidate gene for litter size in sheep. PLoS One. 2021;16(1):e0244408. 10.1371/journal.pone.0244408. PMC7822323 33481819

[71] Li S, Ge F, Chen L, Liu Y, Chen Y, Ma Y. Genome-wide association analysis of body conformation traits in Chinese holstein cattle. BMC Genomics. 2024;25(1):1174. 10.1186/s12864-024-11090-8. PMC11616231 39627684

[72] Liu M, Li B, Huang Y, Yang M, Lan X, Lei C, et al. Copy number variation of bovine MAPK10 modulates the transcriptional activity and affects growth traits. Livest Sci. 2016;194:44–50. 10.1016/j.livsci.2016.09.014.

