# Supplementary material for: Genome-wide association analyses reveal genomics basis for frizzled feather and candidate genes associated with growth traits in Yuexi frizzled feather chicken
Source: Genet Sel Evol. 2026 Nov 12;58:88. doi: 10.1186/s12711-026-01107-z (PMC13757092; doi:10.1186/s12711-026-01107-z)

Breed	Sample location	Feather phenotype	Purpose	Photography
Yuexi Frizzled Feather chicken (YFC, n = 356)	Maoming Country, Guangdong	Frizzle	Resequencing Sanger sequencing	
Qiandongnan Xiaoxiang chicken (QXC, n = 25)	Qiandongnan Miao and Dong Autonomous Country, Guizhou	normal	Resequencing	
Qibainong chicken (QC, n = 60)	Hechi Dahua Country, Guangxi	normal	Resequencing Sanger sequencing	
Bantam chicken (BC, n = 30)	Nanning Country, Guangxi	normal	Sanger sequencing	
Wenchang chicken (WC, n = 30)	Wenchang Country , Hainan	normal	Sanger sequencing	
Huaixiang chicken (HC, n = 30)	Maoming Country, Guangdong	normal	Sanger sequencing	
Qingyuan Ma chicken (QMC, n = 30)	Qingyuan Country, Guangdong	normal	Sanger sequencing	
Huxu chicken (HXC, n = 30)	Huizhou Huiyang Country, Guangdong	normal	Sanger sequencing	

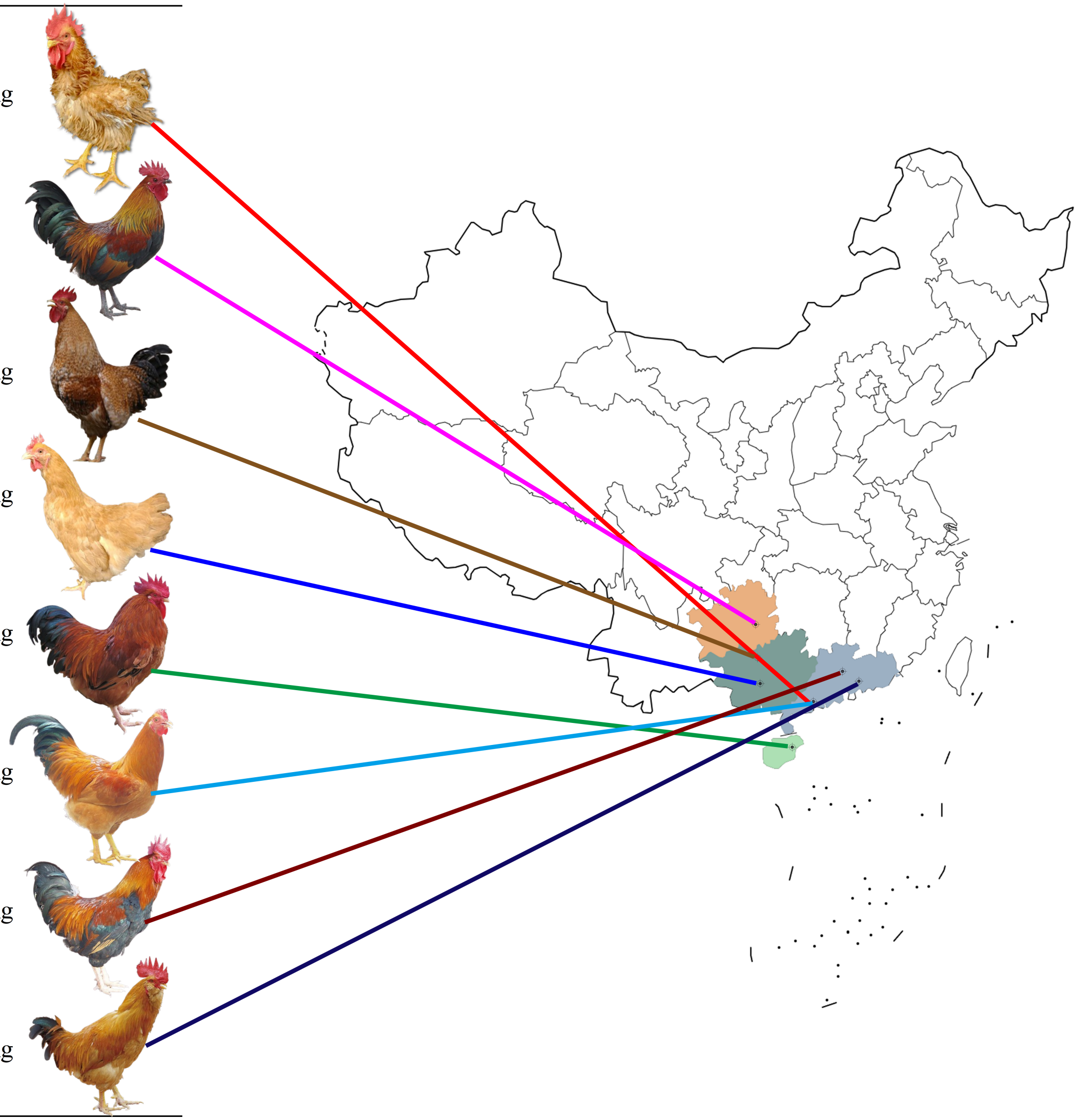

Supplement: Supplementary file 1 [file 12711_2026_1107_MOESM1_ESM.pdf]
