# Supplementary material for: Genome-wide association analyses reveal genomics basis for frizzled feather and candidate genes associated with growth traits in Yuexi frizzled feather chicken
Source: Genet Sel Evol. 2026 Nov 12;58:88. doi: 10.1186/s12711-026-01107-z (PMC13757092; doi:10.1186/s12711-026-01107-z)
Supplement: Supplementary file 2 [file 12711_2026_1107_MOESM2_ESM.pdf]

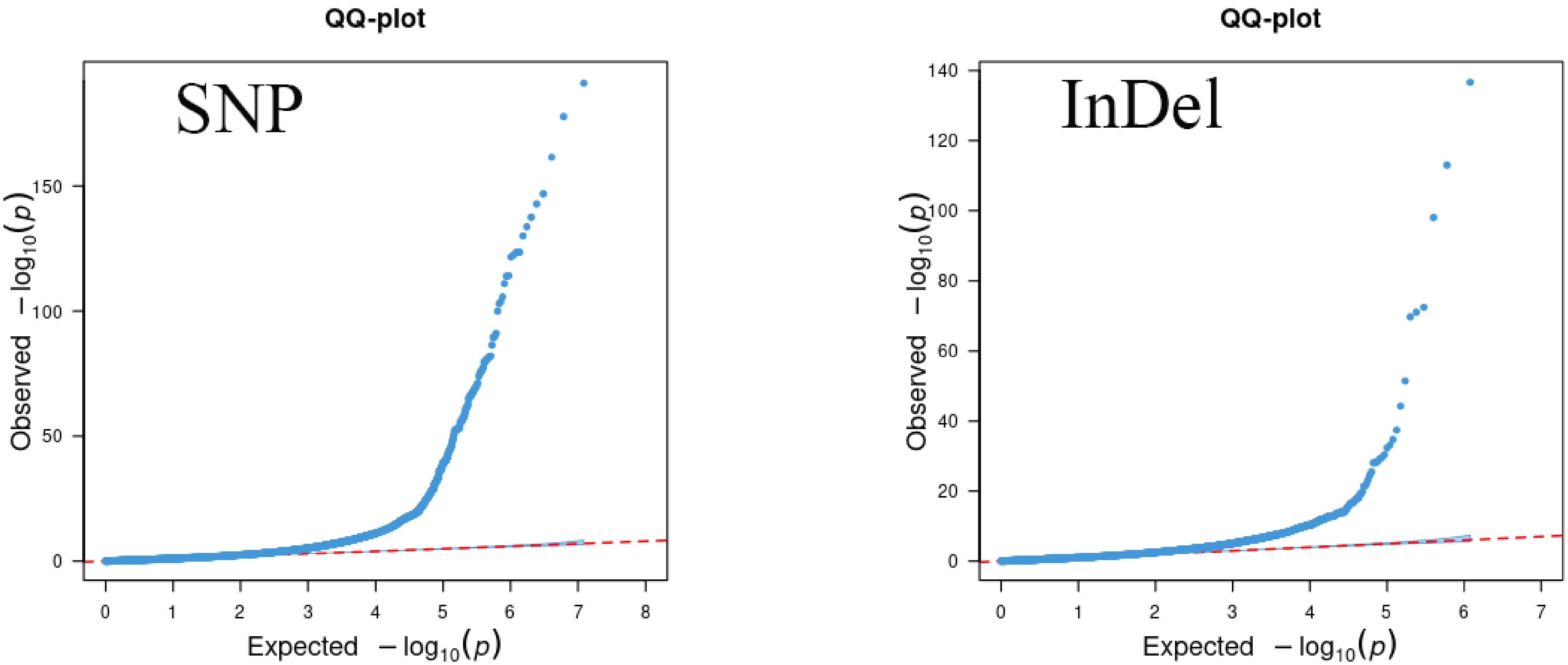
QQ-plot
SNP
Observed −log10(p)
Expected −log10(p)
0
50
100
150
0
1
2
3
4
5
6
7
8
QQ-plot
InDel
Observed −log10(p)
Expected −log10(p)
0
20
40
60
80
100
120
140
0
1
2
3
4
5
6
7
