# Supplementary material for: Genome-wide association analyses reveal genomics basis for frizzled feather and candidate genes associated with growth traits in Yuexi frizzled feather chicken
Source: Genet Sel Evol. 2026 Nov 12;58:88. doi: 10.1186/s12711-026-01107-z (PMC13757092; doi:10.1186/s12711-026-01107-z)
Supplement: Supplementary file 3 [file 12711_2026_1107_MOESM3_ESM.pdf]

KRT75L4

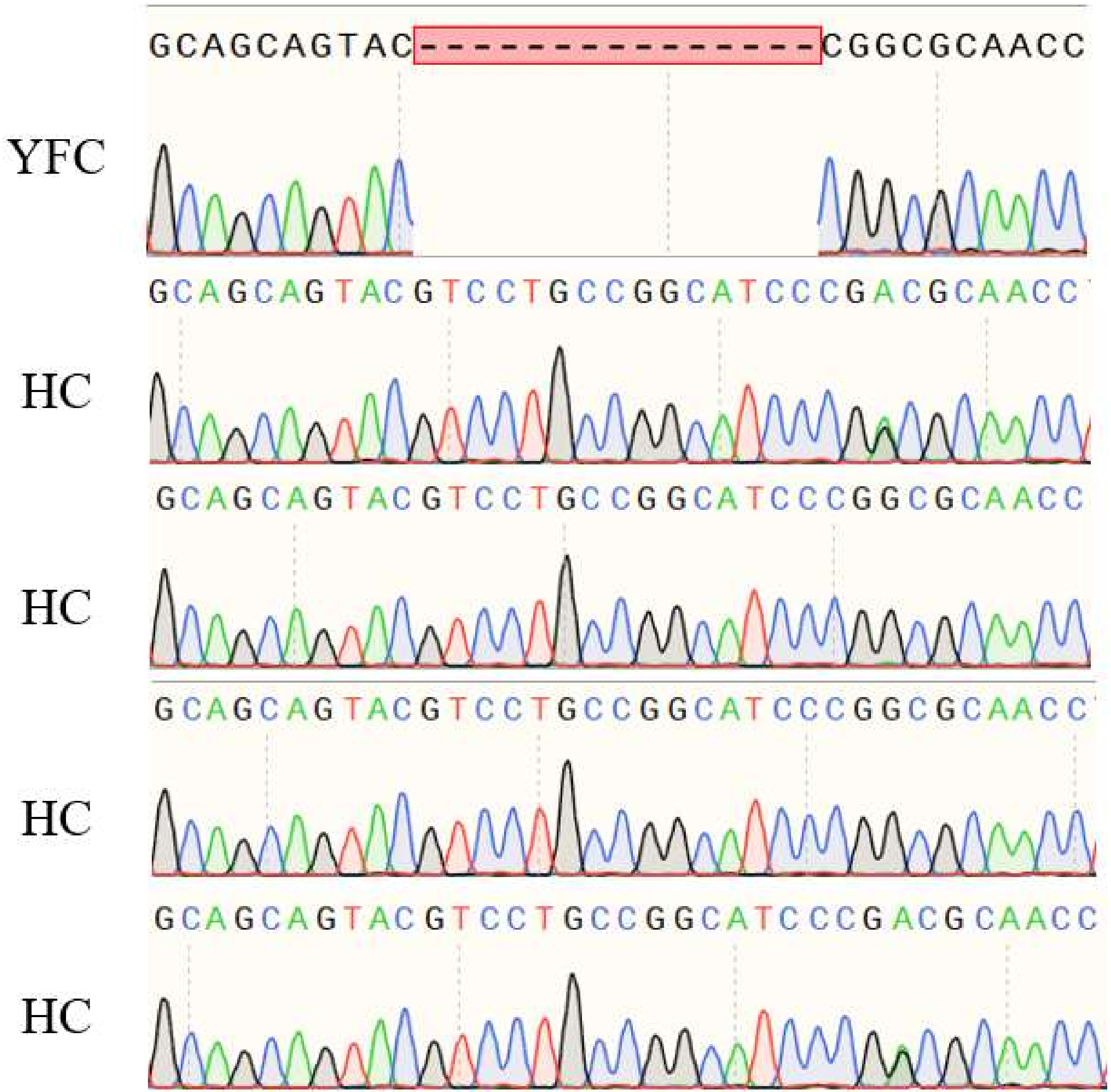
GCAGCAGTAC---------------CGGCGCAACC
YFC
GCAGCAGTACGTCCTGCCGGCATCCCGACGCAACC
HC
GCAGCAGTACGTCCTGCCGGCATCCCGGCGCAACC
HC
GCAGCAGTACGTCCTGCCGGCATCCCGGCGCAACC
HC
GCAGCAGTACGTCCTGCCGGCATCCCGACGCAACC
HC
