# Supplementary material for: Genome-wide association analyses reveal genomics basis for frizzled feather and candidate genes associated with growth traits in Yuexi frizzled feather chicken
Source: Genet Sel Evol. 2026 Nov 12;58:88. doi: 10.1186/s12711-026-01107-z (PMC13757092; doi:10.1186/s12711-026-01107-z)
Supplement: Supplementary file 5 [file 12711_2026_1107_MOESM5_ESM.pdf]

InDel data

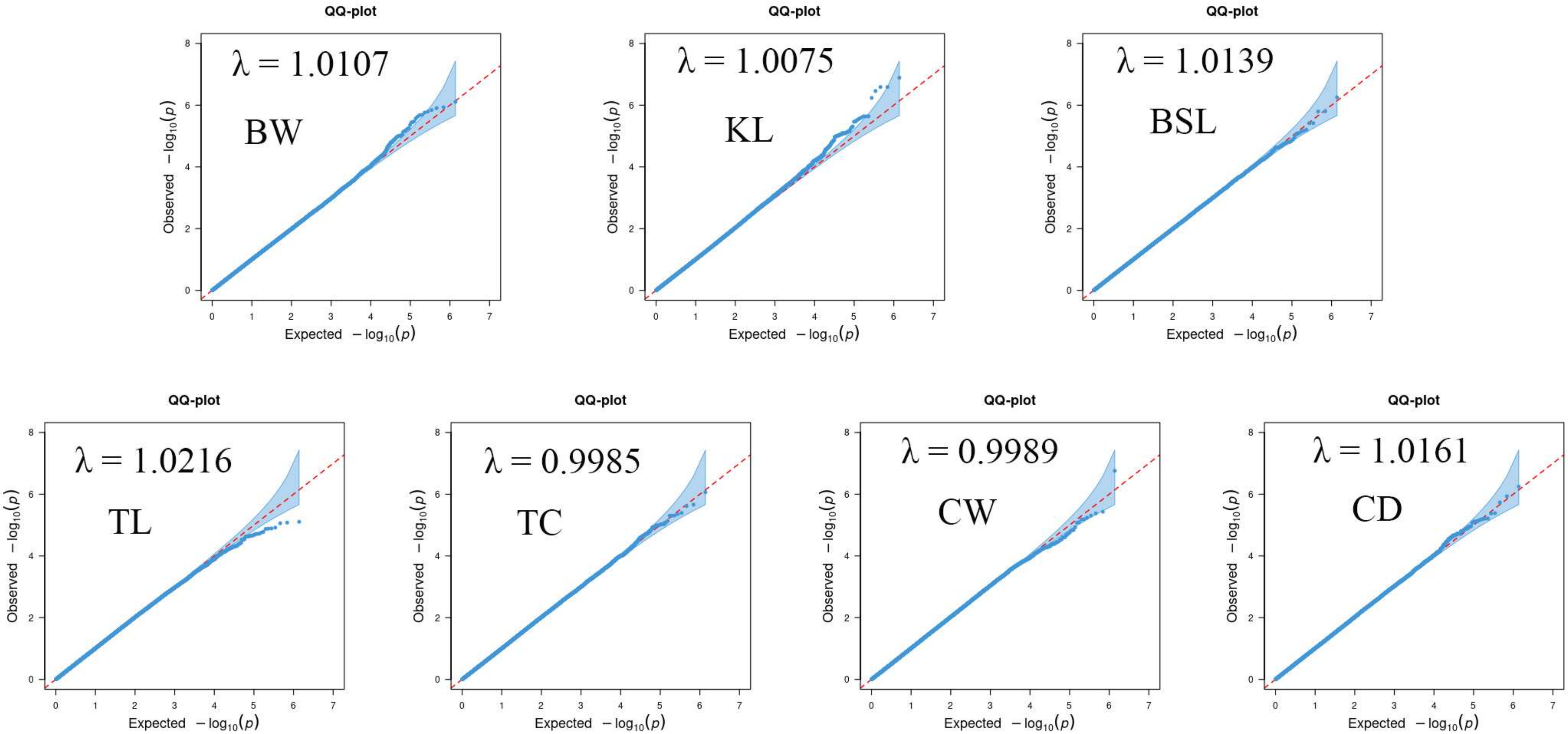

QQ-plot
λ = 1.0107
BW
QQ-plot
λ = 1.0075
KL
QQ-plot
λ = 1.0139
BSL
QQ-plot
λ = 1.0216
TL
QQ-plot
λ = 0.9985
TC
QQ-plot
λ = 0.9989
CW
QQ-plot
λ = 1.0161
CD
Observed −log10(p)
Expected −log10(p)
