# Supplementary material for: Genome-wide association analyses reveal genomics basis for frizzled feather and candidate genes associated with growth traits in Yuexi frizzled feather chicken
Source: Genet Sel Evol. 2026 Nov 12;58:88. doi: 10.1186/s12711-026-01107-z (PMC13757092; doi:10.1186/s12711-026-01107-z)
Supplement: Supplementary file 6 [file 12711_2026_1107_MOESM6_ESM.pdf]

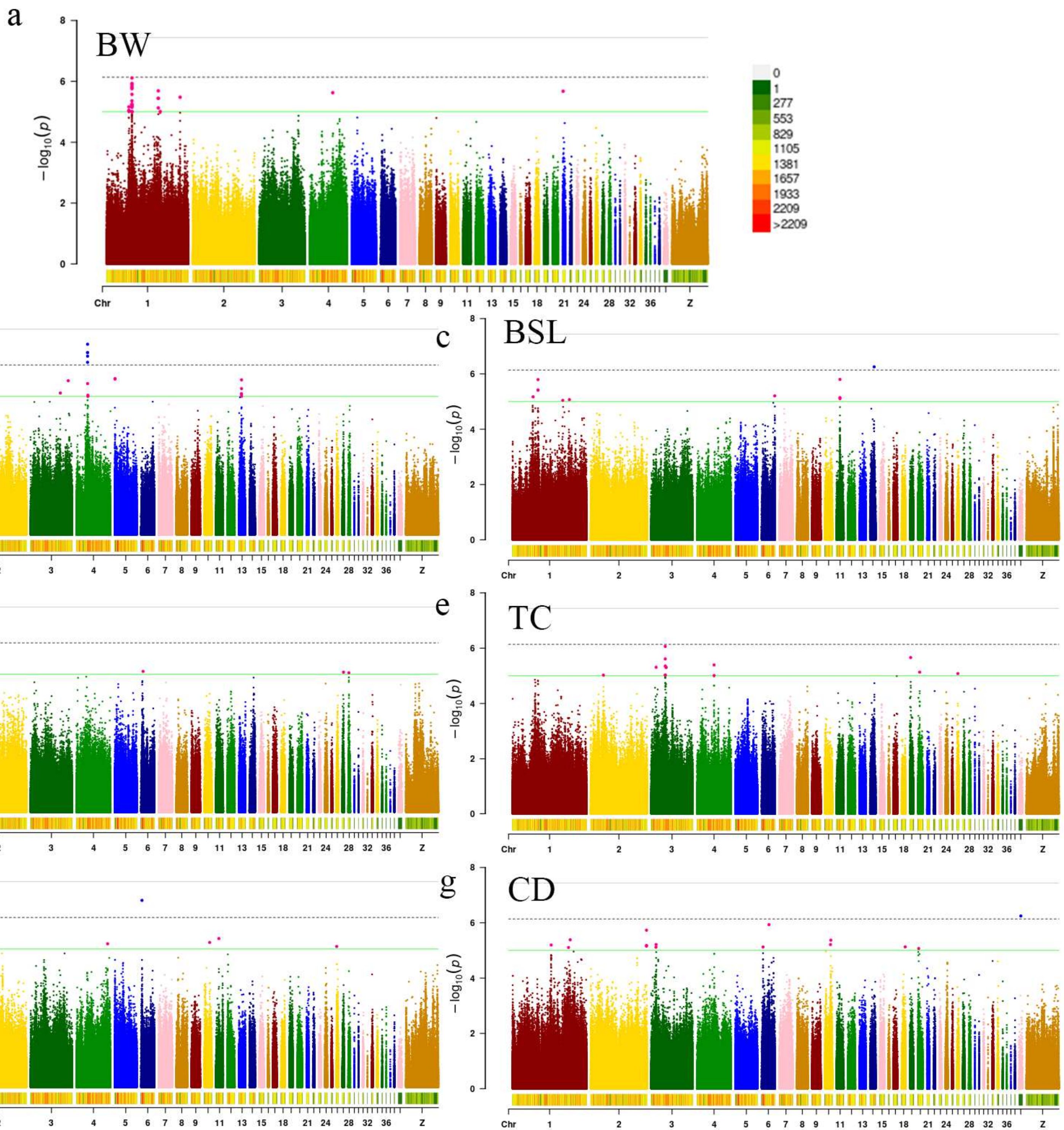
a
BW
b
KL
c
BSL
d
TL
e
TC
f
CW
g
CD
−log10(p)
Chr
1
2
3
4
5
6
7
8
9
11
13
15
18
21
24
28
32
36
Z
0
1
277
553
829
1105
1381
1657
1933
2209
>2209
