# Supplementary material for: Genome-wide association analyses reveal genomics basis for frizzled feather and candidate genes associated with growth traits in Yuexi frizzled feather chicken
Source: Genet Sel Evol. 2026 Nov 12;58:88. doi: 10.1186/s12711-026-01107-z (PMC13757092; doi:10.1186/s12711-026-01107-z)
Supplement: Supplementary file 7 [file 12711_2026_1107_MOESM7_ESM.pdf]

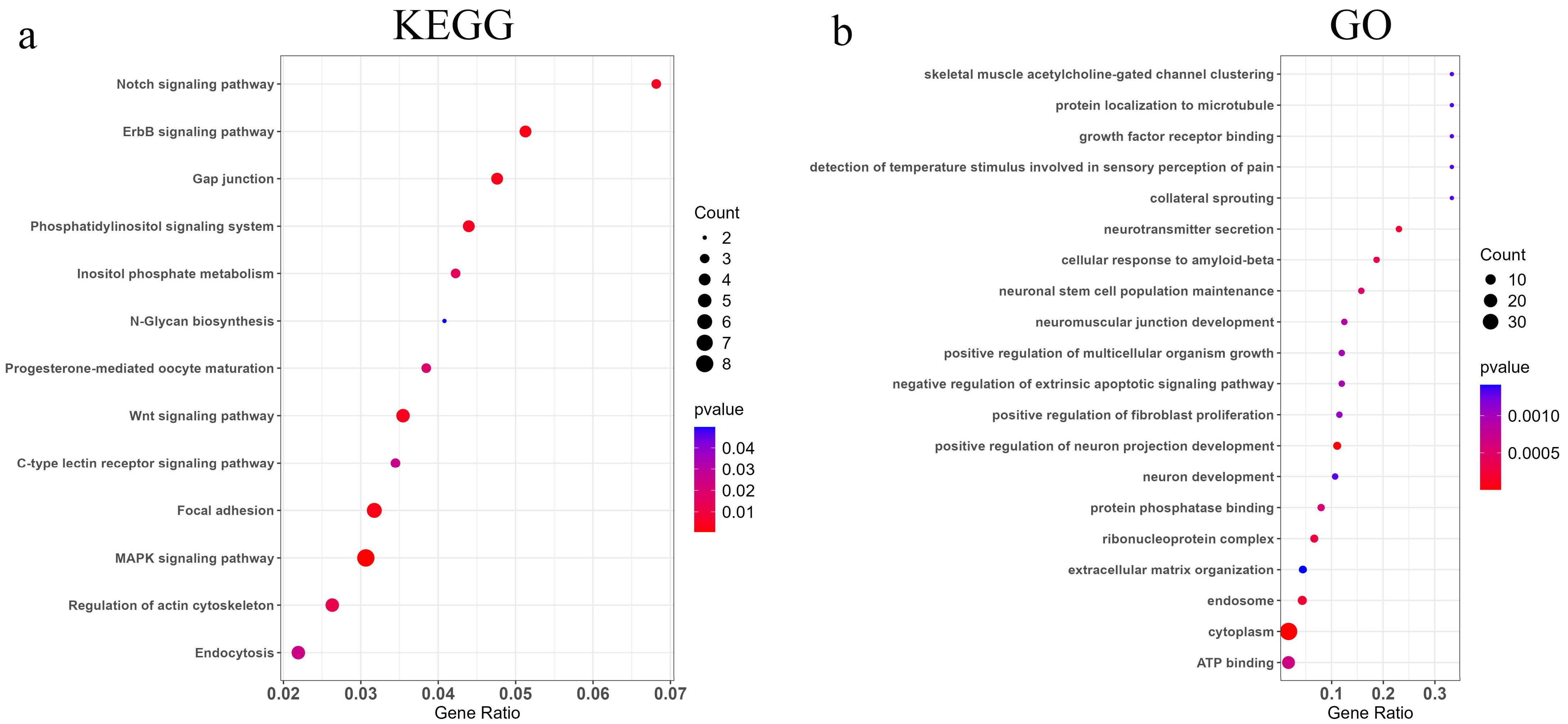
a
KEGG
Notch signaling pathway
ErbB signaling pathway
Gap junction
Phosphatidylinositol signaling system
Inositol phosphate metabolism
N-Glycan biosynthesis
Progesterone-mediated oocyte maturation
Wnt signaling pathway
C-type lectin receptor signaling pathway
Focal adhesion
MAPK signaling pathway
Regulation of actin cytoskeleton
Endocytosis
0.02
0.03
0.04
0.05
0.06
0.07
Gene Ratio
Count
2
3
4
5
6
7
8
pvalue
0.04
0.03
0.02
0.01
b
GO
skeletal muscle acetylcholine-gated channel clustering
protein localization to microtubule
growth factor receptor binding
detection of temperature stimulus involved in sensory perception of pain
collateral sprouting
neurotransmitter secretion
cellular response to amyloid-beta
neuronal stem cell population maintenance
neuromuscular junction development
positive regulation of multicellular organism growth
negative regulation of extrinsic apoptotic signaling pathway
positive regulation of fibroblast proliferation
positive regulation of neuron projection development
neuron development
protein phosphatase binding
ribonucleoprotein complex
extracellular matrix organization
endosome
cytoplasm
ATP binding
0.1
0.2
0.3
Gene Ratio
Count
10
20
30
pvalue
0.0010
0.0005
